# What matters in chronic *Burkholderia cenocepacia* infection in cystic fibrosis: Insights from comparative genomics

**DOI:** 10.1371/journal.ppat.1006762

**Published:** 2017-12-11

**Authors:** Jaroslav Nunvar, Vaclav Capek, Karel Fiser, Libor Fila, Pavel Drevinek

**Affiliations:** 1 Department of Medical Microbiology, 2nd Faculty of Medicine, Charles University and Motol University Hospital, Prague, Czech Republic; 2 Bioinformatics Centre, 2nd Faculty of Medicine, Charles University, Prague, Czech Republic; 3 Department of Paediatric Haematology and Oncology, 2nd Faculty of Medicine, Charles University and Motol University Hospital, Prague, Czech Republic; 4 Department of Pneumology, 2nd Faculty of Medicine, Charles University and Motol University Hospital, Prague, Czech Republic; University of California Davis School of Medicine, UNITED STATES

## Abstract

*Burkholderia cenocepacia* causes severe pulmonary infections in cystic fibrosis (CF) patients. Since the bacterium is virtually untreatable by antibiotics, chronic infections persist for years and might develop into fatal septic pneumonia (cepacia syndrome, CS). To devise new strategies to combat chronic *B*. *cenocepacia* infections, it is essential to obtain comprehensive knowledge about their pathogenesis. We conducted a comparative genomic analysis of 32 Czech isolates of epidemic clone *B*. *cenocepacia* ST32 isolated from various stages of chronic infection in 8 CF patients. High numbers of large-scale deletions were found to occur during chronic infection, affecting preferentially genomic islands and nonessential replicons. Recombination between insertion sequences (IS) was inferred as the mechanism behind deletion formation; the most numerous IS group was specific for the ST32 clone and has undergone transposition burst since its divergence. Genes functionally related to transition metal metabolism were identified as hotspots for deletions and IS insertions. This functional category was also represented among genes where nonsynonymous point mutations and indels occurred parallelly among patients. Another category exhibiting parallel mutations was oxidative stress protection; mutations in catalase KatG resulted in impaired detoxification of hydrogen peroxide. Deep sequencing revealed substantial polymorphism in genes of both categories within the sputum *B*. *cenocepacia* ST32 populations, indicating extensive adaptive evolution. Neither oxidative stress response nor transition metal metabolism genes were previously reported to undergo parallel evolution during chronic CF infection. Mutations in *katG* and copper metabolism genes were overrepresented in patients where chronic infection developed into CS. Among professional phagocytes, macrophages use both hydrogen peroxide and copper for their bactericidal activity; our results thus tentatively point to macrophages as suspects in pathogenesis towards the fatal CS.

## Author summary

The large *Burkholderia cenocepacia* populations which persist in cystic fibrosis lungs during many years of chronic infections have an inherent potential for adaptive evolution. The results provided by comparative genomics are key in understanding the processes involved. Mutational events which have taken place allow us to deductively reconstruct the history of chronic infection and to identify driving forces acting upon the bacteria. Beyond the conventional point mutation analysis of next generation sequencing data, we observed interesting phenomena such as large deletions and transposable element movement which represent another facet of adaptive evolution of *B*. *cenocepacia* during chronic infection. We also found, unexpectedly, that adaptive evolution in *B*. *cenocepacia* strain ST32 affects a set of genes conspicuously different from related species *B*. *dolosa*; these appear to be linked to host immune response. Our study provides clues to the complex puzzle of chronic *B*. *cenocepacia* infection establishment, persistence and outcome in cystic fibrosis.

## Introduction

In cystic fibrosis (CF) patients, thick sputum obturates the airways as a result of CFTR chloride channel defect. This environment is populated by bacterial communities which often include pathogens such as *Staphylococcus aureus*, *Pseudomonas aeruginosa*, *Haemophilus influenzae* and *Stenotrophomonas maltophilia* [1]. Bacteria from *Burkholderia cepacia* complex (Bcc), a monophyletic group within the genus which currently comprises 20 species [2], have emerged during the 1980s as CF pulmonary pathogens [3]. Bcc are generally regarded the most harmful CF pathogens; the infections are associated with significant decline in lung functions and the lingering threat of development of cepacia syndrome [4], a fulminant necrotizing pneumonia with high fatality rate [5].

*B*. *cenocepacia* (representing the former genomovar III) is one of the most prevalent Bcc species encountered in CF infections [6]. Among *B*. *cenocepacia*, two lineages were delineated based on *recA* sequence similarity: IIIA and IIIB [7]. The IIIA lineage, syn. clonal complex 31 (multilocus sequence typing [MLST] CC31), is by far the largest cluster in Bcc MLST database [8], reflecting their frequent isolation from patients. Furthermore, the IIIA lineage was reported to show the most pronounced isolation bias; virtually all MLST sequence types were isolated from clinical sources [9], suggesting tight association with humans or even an ongoing switch from environmental to host-associated lifestyle [10]. Studies clearly demonstrated the particularly destructive nature of IIIA infections in CF patients when compared with other Bcc bacteria [11, 12].

*B*. *cenocepacia* IIIA are known to cause epidemic outbreaks [13]; recently, they were reported to dominate in Serbia (ST856 [14]) and Russia (ST709 [15]). The most notorious epidemic IIIA bacterium is the ET12 clone, a hypervirulent transatlantic strain responsible for large infection outbreaks in Europe and North America [16]. Another globally distributed clone, ST32, was detected in Italy, France, UK and Canada [17]. Czech CF patients were plagued by epidemic spread of this *B*. *cenocepacia* strain (also called CZ1 [18]) in the 1990s. Out of the 57 patients infected; only 15 were alive by 2015 [19].

In this work, we aimed to elucidate the evolution of *B*. *cenocepacia* ST32 during chronic pulmonary CF infections with fatal outcomes. A comprehensive comparative genomic analysis was conducted, covering multiple isolates from multiple patients. The genes and pathways exhibiting parallel evolution and the underlying mutational processes are reported.

## Results

### *B*. *cenocepacia* epidemic strain isolates

**Fig 1:**
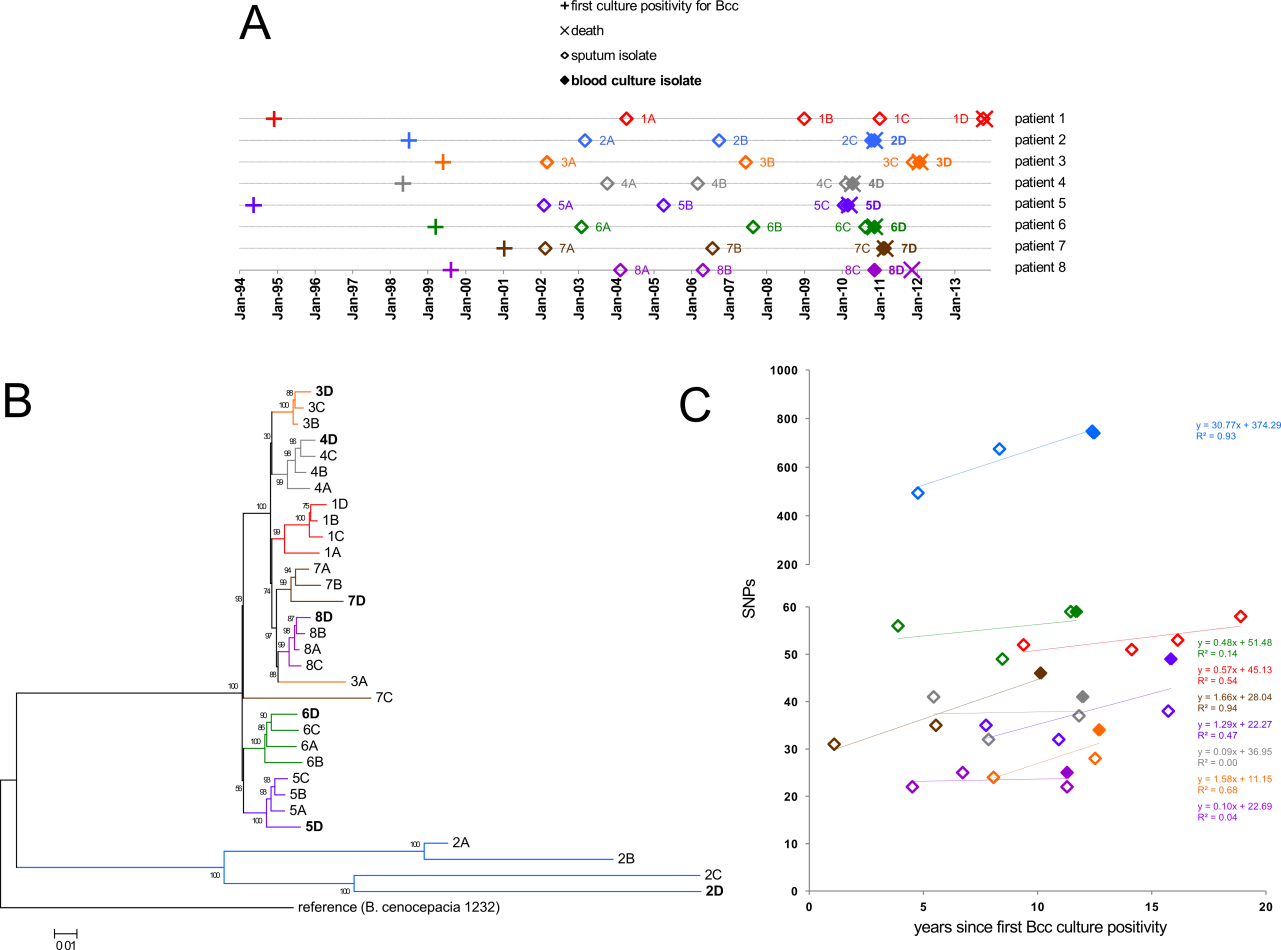
Overview of sequenced *B*. *cenocepacia* ST32 isolates. (A) Sample collection chart. Bacteria were isolated at indicated time points and named by patient IDs (1 to 8) and chronology of isolation A to D (i.e., isolates 1A to 1D, 2A to 2D etc.). Isolates 2C and 2D, 5C and 5D, 7C and 7D, and 8C and 8D overlapped due to their concurrent isolation at the time of CS (see S1 Table for details). (B) Whole-genome phylogeny. Consensus genomic sequences obtained by mapping sequencing reads onto the complete reference genome were used for tree inference. The Maximum-Likelihood tree was constructed from 2,754 variant nucleotide positions using the CSIPhylogeny pipeline [74]. (C) SNP accumulation during chronic infection. SNPs informative of within-patient evolution (see Materials and Methods) were plotted against time elapsed from the first Bcc culture positivity to the point of bacterial isolation.

First, we examined the genetic relationship of the Czech epidemic strain *B*. *cenocepacia* ST32 (CZ1) with other sequenced strains from the *B*. *cenocepacia* group *recA* IIIA (CC31). Whole-genome phylogenetic analysis showed that ST32 was clearly distinct from the ET12 epidemic clone (S1 Fig). To gain insight into ST32 evolution during chronic CF pulmonary infection, a total of 32 isolates were selected for whole-genome sequencing (WGS). The isolates originated from 8 chronically infected patients who ultimately developed cepacia syndrome (CS) (S1 Table). Each patient was represented by 4 chronological isolates. These covered most of the known period of their chronic infection (average 7.9 years). The collection included the first archived sputum isolate, one mid-term sputum isolate and isolates from last sputum sample and blood culture; the two latter isolates corresponded to the time of CS. The only exception was patient 1 where no blood culture isolate at the time of CS was available; thus 1 additional sputum isolate was used to complete the set. Interestingly, patient 8 survived CS episode for one more year, so his last sputum and blood isolates were collected one year before the date of death. All other patients died soon after dates of collection of their CS isolates (Fig 1A). *In silico* MLST analysis of WGS sequences confirmed that all 32 isolates belonged to ST32. The presence of ST32-specific DNA sequence [20] was detected in genomic sequences of all isolates.

From whole-genome sequences of ST32 isolates, phylogenetic tree was constructed (Fig 1B) and the results were assessed with respect to their spatiotemporal relationships. Virtually all isolates (30/32) clustered into patient-specific lineages. This implies that the populations have originated from a single colonization event per patient, followed by subsequent diversification. The intra-patient branching patterns did not always follow the chronology of isolation. For example, blood culture isolates 5D and 7D branched out at the earliest and so did the last sputum isolate 8C. This indicates long-standing co-existence of different subpopulations which evolved from the original colonization.

The isolates accumulated single-nucleotide polymorphisms (SNPs) over the course of infection (Fig 1C). SNP accumulation rate (calculated as linear regression slope) in patient 2 was over an order of magnitude higher than in remaining patients. This was in accordance with markedly increased genomic diversity of isolates retrieved from this patient (Fig 1B). The mutation frequency values determined for isolates 2A-2D corresponded to hypermutator phenotype (4.9 x 10^−6^–8.5 x 10^−6^), while all remaining isolates displayed nonmutator values (3.6 x 10^−9^–5.3 x 10^−8^) (measured and interpreted according to Martina *et al*. [21]) Hypermutability was associated with a 4-nt deletion in the mismatch-repair gene *mutS*. For nonmutator lineages, the weak correlation between SNP numbers and infection duration did not allow for reliable SNP rate calculation (Fig 1C). The value calculated from best linear fit (1.66 SNPs/ year in patient 7) was slightly lower, yet comparable with SNP rates reported during chronic pulmonary infection for other CF pathogens such as *B*. *dolosa* (2.1 SNPs/year [22]), *B*. *multivorans* (2.4 SNPs/year [23]), *P*. *aeruginosa* (2.7 SNPs/year [24]) and *Burkholderia pseudomallei* (3.6 SNPs/year [25]).

### ST32 genome dynamics during chronic infection

**Fig 2:**
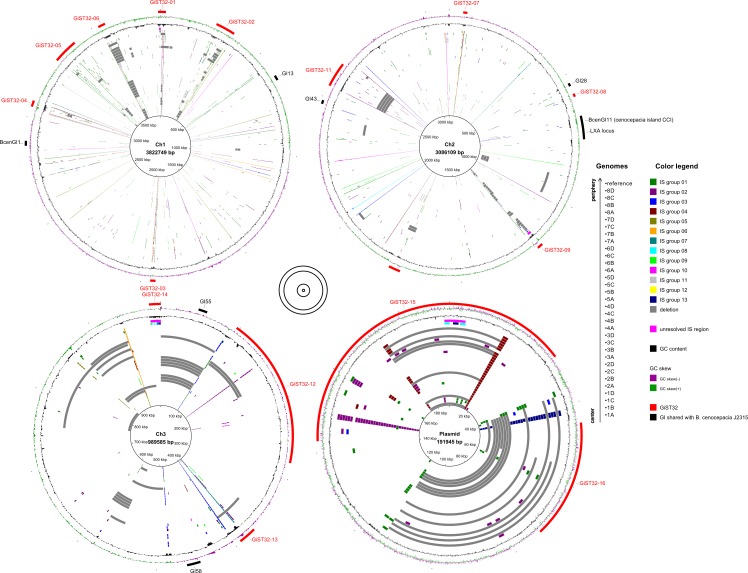
Insertion sequences (IS), genomic islands (GI) and large-scale deletions (LSD) in ST32 isolates. The inner circles denote particular isolates´ genomes and reference genome, ordered as indicated. IS insertions and LSDs over 10 kb in size are colored as explained in the legend. The outer circles denote GC skew, GC content and GIs as explained in the legend. The four replicons are not plotted to scale; their relative sizes are denoted in the middle. Visualizations were carried out in BRIG [82]. For source data, see S3–S6 Tables.

Mapping of sequencing reads to reference genome detected many cases when large portions of reference genome showed missing coverage. Most of these large-scale deletions (LSDs) were not shared among patients and thus represented regions of ST32 genome lost during diversification in chronic infection (Fig 2, S3 Table). The putative genomic islands (GIs) specific for ST32 epidemic clone were detected by pairwise comparison of its genomic sequence with ET12 isolate *B*. *cenocepacia* J2315 [10] and termed GiST32-01 to GiST32-16 (S4 Table). Analysis of possible association between LSDs and GIs revealed important differences among the four replicons (Fig 2). LSDs on essential replicons (chromosomes 1 and 2) were localized almost exclusively in ST32 GIs. Different pattern was observed with non-essential replicons (chromosome 3/megaplasmid [26] and plasmid [27]) where LSDs removed significant portions of DNA regardless of GI positions, in accordance with the dispensable nature of these replicons. Although numerous LSDs were present in non-essential replicons, no complete loss of either chromosome 3 or plasmid was detected among our ST32 dataset. On the contrary to ST32-specific GIs, GIs that were shared with *B*. *cenocepacia* J2315, including the previously characterized cenocepacia island *cci* [28] and the *lxa* locus [29], were not affected by LSDs at all. This, in conjunction with their conservation between the two epidemic strains, corroborates their functional importance.

Preliminary inspection revealed transposase genes at the borders of many LSD regions, which prompted us to look into ST32 transposable elements (insertion sequences, ISs) in more detail. 13 groups of ISs present at two or more copies in reference genome were detected, and their abundance in complete *B*. *cenocepacia* IIIA genomes was assessed. Strikingly, the most numerous IS group 01 (53 copies in ST32 reference genome) was found to be absent from genomes of all closely related *B*. *cenocepacia* strains, indicating its very recent acquisition and transposition burst (S5 Table). Insertions of all IS groups were analyzed *in silico* in the WGS dataset of ST32 isolates using ISMapper [30] (Fig 2, S6 Table). Numerous new insertions were detected in the dataset; the most abundant IS (group 01) was also the most mobile. Many cases of intra-patient, lineage-specific IS insertions were observed, corroborating their relationships inferred from whole-genome phylogeny (Fig 1B). IS insertions were substantially enriched in GIs in comparison with the rest of the genome (Fig 2, S6 Table).

### Parallel (inter-patient) evolution

Detailed examination of ISs in the ST32 WGS dataset revealed that several genes experienced multiple, independent IS insertions during chronic infection. When analyzed for the presence of conserved domains [31], metal-related functions were predicted. These genes (TQ36_15160, TQ36_15180, TQ36_25385 and TQ36_35715/*copD*; S2 Fig) were located within ST32-specific GIs and were surrounded by other metal-related genes, each on a different replicon. In addition to IS insertions, deletions of varying sizes were detected upon detailed analysis of mapped sequencing reads (S2 Fig). The extent of parallelism was remarkable: each gene was inactivated in at least 6 out of total 8 patients. The plasmid-located copper resistance gene *copD* (TQ36_35715) and neighboring copper-related genes were affected by the greatest number of deletions (8 independent events; S2 Fig). Together, parallel IS insertions and deletions suggest that inactivation events were positively selected during chronic infection in CF sputum.

**Fig 3:**
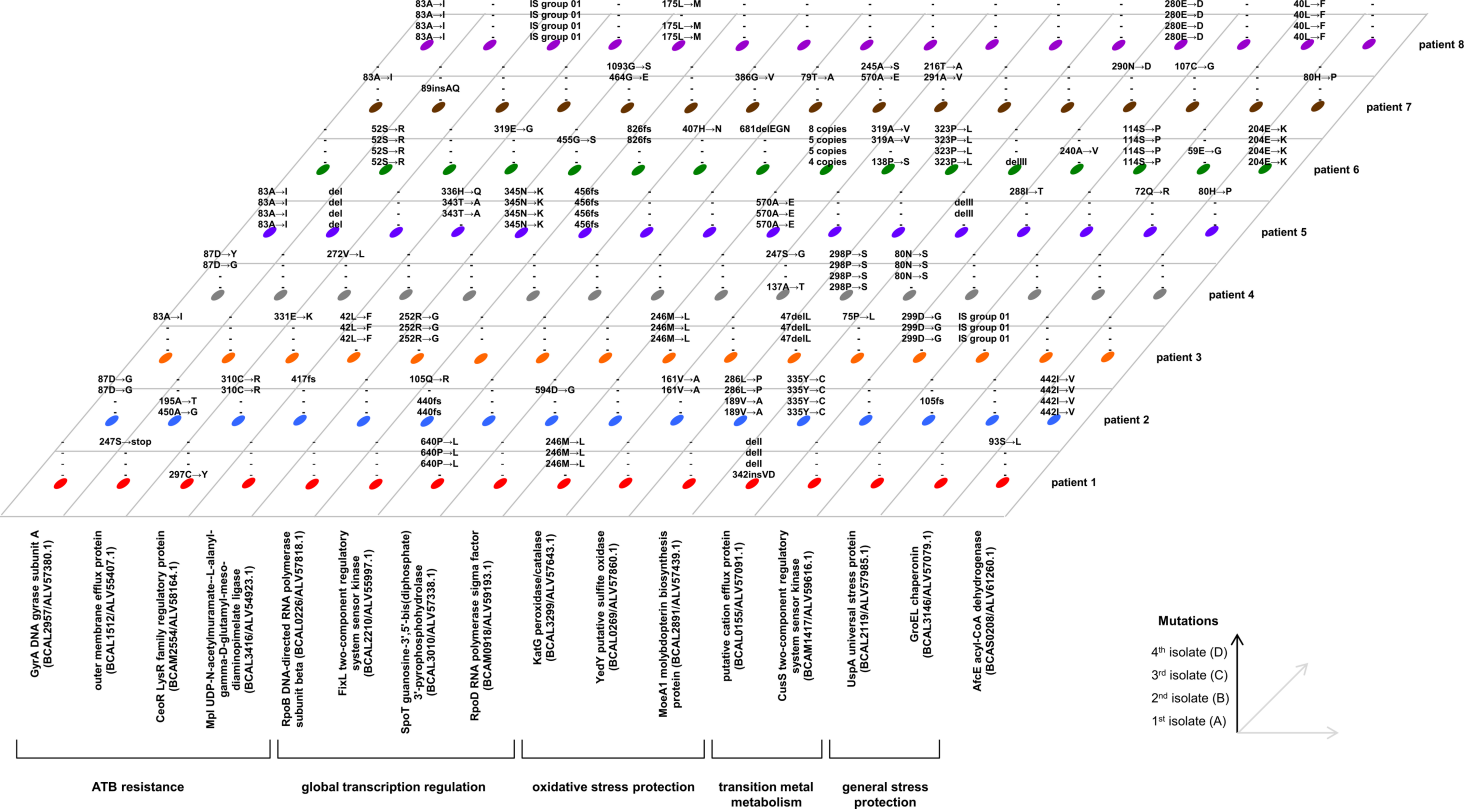
3D-plot of parallel mutations among 8 patients with chronic ST32 infections resulting in CS. All genes where nonsynonymous substitutions occurred in at least 1 isolate from at least 3 patients are depicted. Reported mutations have arisen during within-patient evolution, *i*.*e*. are missing in ancestral ST32 genotype which initially colonized the patient (inferred from WGS phylogeny, Fig 1B).

In the search of further evidence for convergent evolution among bacteria from different patients, we analyzed point mutations, *i*.*e*. single nucleotide polymorphisms (SNPs) and short insertions and deletions (indels). We focused on intragenic, nonsynonymous mutations which have arisen during diversification of ST32 populations after initial colonization of patients’ lungs. Analysis of SNP distribution showed that within the ST32 WGS dataset, genes harboring 2 or more independent SNPs were overrepresented in comparison with neutral model (S3 Fig); ≥3 SNPs per gene were not predicted to occur in the neutral model. Genes were thus considered to have undergone parallel evolution if they received 3 or more independent nonsynonymous mutations. 16 out of 6,939 genes present in ST32 genome met this criterion (Fig 3). Multiple nonsynonymous mutations were typically present (4.75 unique mutations per gene on average), while synonymous mutations (a measure of “background” mutation rate) were absent in a great majority (13/16) of the investigated genes. These values deviate strongly from the theoretical frequencies calculated by Dillon *et al*. for *B*. *cenocepacia* (72% nonsynonymous vs. 28% synonymous mutations [32]), indicating positive selection acting upon the genes.

Based on available literature, genes harboring parallel nonsynonymous mutations (Fig 3) were grouped into 5 functional classes: antibiotic resistance (4 genes), global transcription regulation (4 genes), oxidative stress protection (3 genes), transition metal metabolism (2 genes) and general stress protection (2 genes); *afcE*, whose role in *B*. *cenocepacia* physiology is complex [33], was the only uncategorized protein. Antibiotic resistance genes and global transcription regulators were previously described to undergo parallel evolution in *B*. *dolosa* [22]; mutations in *gyrA* affected the same amino acid positions and were associated with high-level levofloxacin resistance (S1 Table). In contrast, genes predicted to function in oxidative stress protection and metal metabolism have not yet been reported to be subject to selection during chronic pulmonary infection in other CF pathogens (see Discussion).

KatG (BCAL3299, also called KatB), the major hydrogen peroxide-detoxifying enzyme with hybrid catalase/peroxidase activity [34, 35], was among the three proteins connected with oxidative stress protection. Mutations in KatG were restrained to three sites; two identical mutations (246M→L and 570A→E) have arisen independently in two patients each, suggesting a tightly limited parallel evolution. Furthermore, the region containing the *katG* gene was multiplicated in isolates from patient 6; as deduced from sequencing read coverage, the copy numbers increased from 4 to 8 during the progress of infection (Fig 3). Together, these results indicate strong selection acting upon KatG. In contrast, other catalase genes present in ST32 genome (homologs of BCAL3477, BCAM0181, BCAM0931 and BCAS0635 in *B*. *cenocepacia* J2315) did not harbor multiple mutations. Another oxidative resistance protein found to be under parallel evolution, YedY (BCAL0269), is a methionine-sulfoxide reductase which repairs periplasmic proteins [36]. In *Escherichia coli*, YedY repairs proteins damaged by hypochlorous acid, a molecule which also induces the expression of *yedY* [36]. YedY requires a molybdopterin cofactor for catalysis; it is the only molybdopterin-dependent enzyme in *E*. *coli* which uses this cofactor in its nucleotide-free form [37]. Since MoeA1 (BCAL2891) catalyzes precisely the final step in the biosynthesis of nucleotide-free molybdopterin, it was regarded to belong to the same functional category as YedY.

Among the most mutated genes, two were related to transition metal metabolism. BCAL0155 is a member of cation diffusion facilitator family of divalent metal efflux transporters [38] whose substrate specifity has not yet been determined. The sensory kinase CusS (BCAM1417) senses periplasmic copper and through its cognate response regulator activates copper resistance mechanisms [39]. Importantly, the genes experiencing rapid inactivation by deletions and IS insertions were also predicted to perform metal-related functions (see above).

### Within-patient population analysis of genes under parallel evolution

Given the rapid convergent evolution of genes involved in transition metal metabolism and the functional importance of oxidative stress defense, all genes under parallel evolution belonging to these two categories (together with an essential gene *rpoB*) were subjected to further analysis, which aimed to unravel the true extent of genetic parallelism in *B*. *cenocepacia* ST32 chronic CF infections.

We investigated 12 sputum samples collected from 12 patients chronically infected with ST32 (Table 1). These patients were all clinically stable at the time of sputum collection and have not developed CS (“non-CS patients”). The genes were PCR-amplified from total sputum DNA. Further processing differed according to the type of mutations observed in the WGS dataset: 6 genes affected by point mutations (Fig 3) were subjected to deep population sequencing (DPS), while PCR amplicons of 4 genes affected by structural variation (S2 Fig) were sized by agarose electrophoresis.

**Table 1:** Parallel mutations in *B*. *cenocepacia* ST32 sputum populations from 12 chronically infected, non-CS patients.

**Patient**	**Sex**	Duration of chronic infection	Point mutations, indels (% ST32 population)^*^						Structural variations^§^			
			MoeA1	YedY	KatG	RpoB	CusS	BCAL0155	TQ36_15160	TQ36_15180	TQ36_25385	CopC/CopD
**I**	M	16 yrs.	• **72Y→H (98)** • **219Y→H (15)** • **383G→D (4)**	• **10V→A (6)** • **116V→A (53)** • **143V→A (15)** • **165E→G (12)** • **231N→S (5)** • **239V→A (2)** • **321M→V (14)**	• -	• 203V→A (3) • 352Y→H (3) • 398E→K (17)	• -	• **370F→S (n.d.)** • **del I (n.d.)** • **del II (n.d.)** • **del V (n.d.)**	**0**	**0**	WT	WT
**II**	F	17 yrs.	• **del I (100)** • **391fs (3)**	• -	• **189L→R (100)**	**• 254E→K (75)**	• -	• **220fs (7)** • **239D→V (2)** • **del III (n.d.)** • **del V (n.d.)**	WT	**IS**	**IS**	WT
**III**	M	20 yrs.	• 250L→R (3) • 20del II (18)	**• 213N→S (100)**	• -	• **177F→L (75)** • **348D→E (9)**	• 193A→V (11)	• del VI (7)	WT	WT	**WT+IS**	**WT+IS**
**IV**	M	14 yrs.	• 241G→S (9) • 277D→N (9) • 304C→R (2)	• 225Q→R (3)	• -	• -	• -	• **82G→S (24)** • **161W→R (24)** • **270H→R (14)**	**WT+IS**	**WT+IS**	**IS**	**WT+IS**
**V**	F	18 yrs.	**• 59F→L (88)**	• 165E→G (3) • 325M→V (9)	• -	• 129M→V (3) • 341T→A (2) • 343Y→C (15)	• -	• **82G→D (15)** • **214L→P (13)** • **216D→G (18)** • **268E→K (17)** • **270H→R (16)** • **290L→P (8)**	**0**	**0**	**WT+IS**	WT
**VI**	F	22 yrs.	• -	• -	• -	**• 464G→E (100)**	• **241D→N (97)**	• -	WT	**0**	WT	**0**
**VII**	M	16 yrs.	**• del III (100)**	**• 172P→L (99)**	• -	**• 351P→L (99)**	• 354T→M (3)	• **244R→C (77)** • **253delLVDAHI (2)** • **338fs (5)**	**IS**	WT	**IS**	WT
**VIII**	M	20 yrs.	• **59F→L (23)** • **del IV(73)** • **del V (43)** • **323P→L (8)**	• **8R→C (3)** • **21I→V (2)** • **159I→N (9)** • **162P→A (4)** • **233A→V (30)** • **319A→V (13)** • **326D→N (10)**	• -	• **273K→E (3)** • **409M→V (76)**	• 146C→Y (4) • 431M→I (3)	• **216D→G (3)** • **262P→L (24)** • **del IV (45)**	**WT+IS**	WT	**WT+IS**	WT
**IX**	F	12 yrs.	**• 142fs (95)**	• **22T→A (24)** • **247S→N (6)** • **325M→V (27)**	• -	• -	• 292M→V (48)	• **del III (n.d.)** • **del V (n.d.)**	**IS**	WT	**IS**	**0**
**X**	F	9 yrs.	**• 266V→A (99)**	**• 165E→G (100)**	• -	• 345N→S (6)	• **27G→R (20)** • **36L→P (16)** • **287V→A (5)** • **337H→Q (2)** • **357L→P (9)** • **380L→P (59)** • **433Gdel (16)**	• **295P→L (55)** • **343D→G (22)**	**0**	**0**	WT	WT
**XI**	M	17 yrs.	**• 298P→S (100)**	**• 129D→N (100)**	• **189L→R (100)**	• -	**• 4S→L (98)**	• del V (7)	**0**	**0**	**IS**	**WT+IS**
**XII**	M	19 yrs.	• **99G→C (91)** • **271delVSVG (7)**	**• 2W→C (91)**	• -	**• 455G→S (83)**	329E→K (9)	**• del V (100)**	**0**	**0**	WT	WT
**Total patients affected**^**$**^			9	8	2	6	3	9	9	8	8	5
			**oxidative stress protection**				**transition metal metabolism**					

*Point mutations and small indels in ST32 population were determined by DPS; their frequencies are denoted in parentheses (average values for two replicate experiments performed on the same sputum DNA template, cut-off: 2%). Identical deletions between repeats are denoted by the same Roman numerals. Population frequencies of overlapping deletions could not be determined (n.d.).

^§^Structural variations were determined by agarose gel electrophoresis: 0 –no PCR amplification (deletion), IS–PCR fragment of increased length (IS insertion), WT–PCR fragment of length corresponding to intact gene, WT+IS–mixed population (PCR fragments of both lengths).

^$^Sum of patients with point mutations and indels detected in over 50% population, or structural variations detected by electrophoresis. The corresponding results are in bold.

The composition of detected mutations allowed us to predict the type of selection for all investigated genes (Table 1). In BCAL0155 and *moeA1*, short deletions and frameshifts were present in addition to nonsynonymous substitutions, indicating selection for inactivation. In contrast, only nonsynonymous substitutions were detected in *cusS*, *katG*, *yedY* and the control essential gene *rpoB* (BCAL0226), suggesting selection for more subtle functional alterations of the encoded proteins. MoeA1 appeared as the first-line protein to mutate; typically, mutations were fixed or nearly-fixed in ST32 population, while other genes were mutated only in a subset of the same population. Indirectly, this implies substantial selective advantage of MoeA1 inactivation which had enabled the mutated bacterium to outcompete its direct ancestors and rise to fixation before other mutations appeared. For other proteins, different pattern was observed in some ST32 populations; up to 7 different mutations coexisted in a patient, typically covering the entire ST32 population (as inferred from their summed frequencies) (Table 1). Selective pressure for mutations in genes undergoing convergent evolution might therefore drive genetic diversification of pulmonary ST32 populations.

Electrophoretic analysis of metal-related genes located on ST32 GIs (S2 Fig) confirmed their frequent inactivation during ST32 chronic infection (Table 1). Since PCR can neither detect deletions spanning amplified fragment nor reveal deleterious point mutations if present in presumably heterogeneous ST32 populations, the reported extent of inactivation is likely to be underestimated.

**Fig 4:**
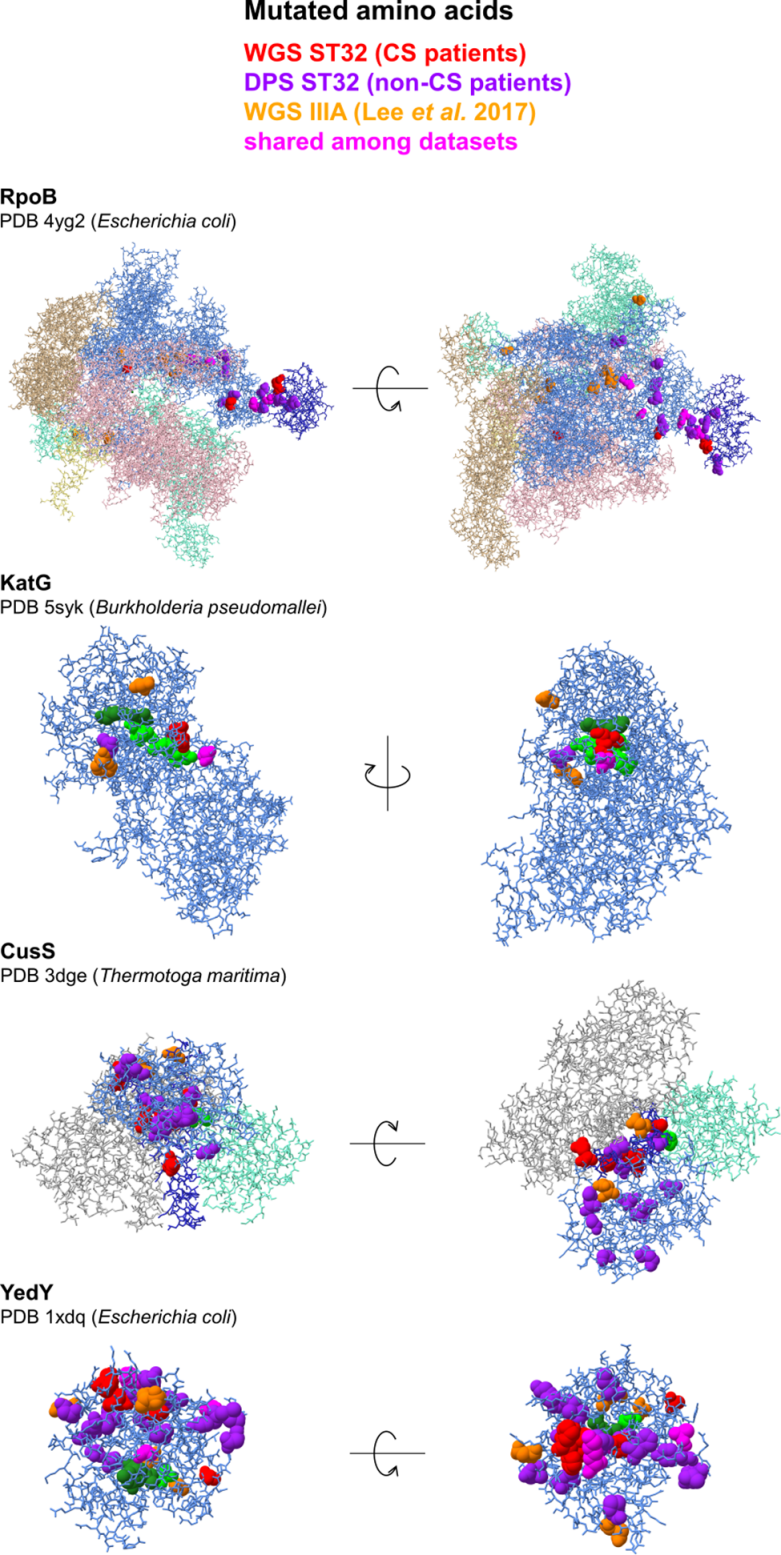
Localization of mutations in proteins under parallel evolution during chronic infection. Protein chains under parallel evolution are denoted in light blue, their functional domains are denoted in dark blue (DHp in CusS, βi4 in RpoB). Cofactors are denoted as forest green spheres, catalytic and active site amino acids are denoted as lime green spheres. Amino acids homologous to residues affected by mutations during chronic Bcc infection (S10 Table) are denoted as spheres and colored as explained in the legend (for ST32 WGS data, see Fig 3; for ST32 DPS data, see Table 1; for IIIA WGS data, see S9 Table). Visualizations were carried out in Chimera [83].

To further characterize the wealth of mutations detected to have arisen during chronic ST32 infection, we mapped them to available 3D structures of homologous proteins (RpoB, CusS, KatG and YedY) (Fig 4). For reference, mutations extracted from genomic sequences of a diverse dataset of *B*. *cenocepacia* IIIA isolates representing various clonal lineages were included (S9 Table [40]). Mutations in YedY appeared scattered through the structure. Most mutations in the catalytic subunit of RNA polymerase (RpoB) localized into a distinct cluster which overlapped or neighbored with the βi4 region (also called dispensable region I) [41], whose function is yet to be established. Curiously, we found dataset-dependent mutation patterns. In KatG, all 3 residues mutated in isolates from CS patients (WGS dataset) were in direct contact with the catalytic MYW cofactor or arginine switch [42], while mutations from both other datasets mapped to residues positioned further apart from the cofactor. Mutations in the sensory kinase CusS also showed different distribution of mutations. Residues mutated in CS patients resided exclusively in the DHp (dimerization and histidine phosphotransfer) domain [43] (S10 Table) and its immediate vicinity. On the other hand, mutations from non-CS patients localized randomly throughout the protein, without preference for DHp.

### Mutation-to-phenotype relationships

**Fig 5:**
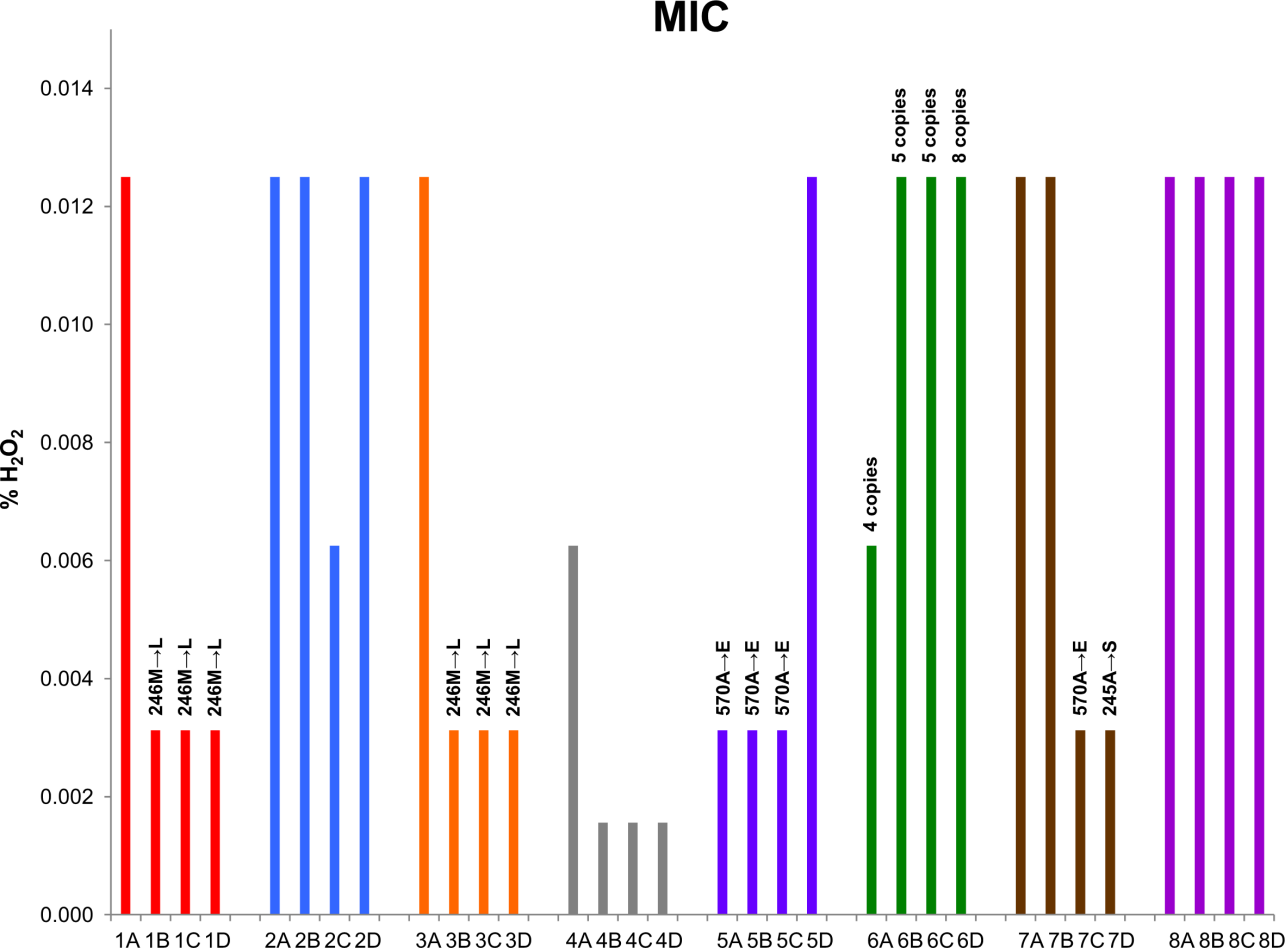
Sensitivity of ST32 isolates to hydrogen peroxide. Minimum inhibitory concentrations (MIC) towards H_2_O_2_ were recorded after aerobic growth in Luria broth at 37°C for 24 h. MIC values were constant during 3 biological replicates.

Finally, we examined if the presumed adaptive mutations resulted in corresponding phenotypic changes. We compared *in vitro* susceptibilities of all ST32 isolates which were genotypically characterized by WGS (Fig 1) towards following substances: copper (II) chloride (CuCl_2_), sodium hypochlorite (NaClO) and hydrogen peroxide (H_2_O_2_). All isolates exhibited uniform level of resistance to both CuCl_2_ and NaClO (8 mM and 0.0625%, respectively), despite the multitude of mutations affecting repair of NaClO-induced oxidative damage (*yedY*, *moeA1*) and metabolism of copper (*cusS*, *copCD*). In sharp contrast, H_2_O_2_ resistance varied significantly; 8-fold range of MIC values was observed (Fig 5). All point mutations in *katG* were associated with decreased resistance, implicating impaired detoxification of H_2_O_2_ by mutant KatG. Furthermore, a trend of MIC decrease during chronic infection was observed in 5 out of 8 patients (Fig 5).

## Discussion

*Burkholderia cepacia* complex bacteria (Bcc) remain the most feared CF pathogens. Virulent lineages like *B*. *cenocepacia recA* group IIIA [7] are of particular concern. Due to huge population size in CF sputum and both intrinsic and rapidly arising acquired multiresistance to antibiotics, *B*. *cenocepacia* infections are virtually impossible to eradicate. To fight chronic Bcc infections, it is urgent to obtain comprehensive knowledge about their pathogenesis. A multitude of virulence factors were discovered in Bcc [44–46]. Furthermore, evolution of Bcc during chronic pulmonary infection might create variability underlying its pathogenic progress, in the worst-case scenario resulting in a fatal outcome (CS). Comparative genomics and analysis of genetic parallelism provide a powerful approach for detection of processes undergoing adaptive changes [47–49]. Our knowledge on evolution and selection during chronic Bcc infection remains scarce, especially for the *B*. *cenocepacia* species. Comprehensive comparative genomic studies have so far been performed only on *B*. *dolosa* and *B*. *multivorans* [22, 23, 50], two species distantly related to *B*. *cenocepacia* within the Bcc [51] which are far less globally distributed (*B*. *dolosa*) or have generally lower virulence potential (*B*. *multivorans*). We thus conducted an evolutionary study of epidemic strain *B*. *cenocepacia* ST32 based upon genomic analysis of multiple isolates originating from multiple patients who succumbed to CS.

### Rapid genomic evolution of epidemic clone ST32

A particular insertion sequence (IS group 01) detected in the epidemic strain ST32 showed conspicuous characteristics. With over 50 copies in reference ST32 genome, this IS was more abundant than any other IS in Bcc genomes investigated [52]. Interestingly, IS group 01 was absent in all other clones within the IIIA lineage (S5 Table). This implies that IS group 01 was acquired very recently during divergence of the ST32 clone and has since then undergone excessive transposition. Similar cases of IS proliferation were detected in flexible genomes of bacteria rapidly adjusting to new lifestyles [53], for example *Shigella* spp. adapting to human intracellular environment [54] or *Burkholderia mallei* becoming a host-specific obligate pathogen [55]. Expansion of IS elements is recognized to be one of general mechanisms underlying evolution of bacterial species which recently diversified from a single clone into highly virulent human-restricted pathogens [56]. Interestingly, among *B*. *cenocepacia* genomes studied by Graindorge *et al*. [52], the hypervirulent clone ET12 (isolate J2315) was found to harbor the largest number of IS copies. Furthermore, in both ST32 and ET12, genomic islands specific for each of the clones were substantially enriched in IS insertions (Fig 2, [52]). This suggests that IS proliferation is a general phenomenon in evolution of virulent lineages of *B*. *cenocepacia*. Our analysis of ST32 isolates also detected copious IS insertions which occurred during chronic CF infection (S6 Table), directly demonstrating the extent of genetic plasticity conferred by these mobile elements. The second most numerous IS group 2 (syn. IS*Bcen20*) was previously noted to be highly mobile under conditions of oxidative stress [57].

Surprisingly, we observed many LSDs to arise in ST32 genomes during chronic CF infection, preferentially affecting GIs and nonessential replicons. Investigating the mechanism behind LSD formation, we often detected IS at the termini of deleted regions (S2 Fig, S6 Table). Recombination between two congruent IS copies was reported as a major mechanism generating spontaneous deletions in *E*. *coli* experimental evolution [58, 59]; apparently, LSDs in chronic ST32 infection arise by the same mechanism. A question is why LSDs have occurred in ST32 so frequently (Fig 2, S3 Table). Some genes located on GIs clearly experienced convergent inactivation, either by IS insertions or LSDs (S2 Fig, Table 1), raising the possibility that some LSD might be adaptive, *i*.*e*. subject to positive selection. In *E*. *coli*, deletions between two IS copies were found to occur at high frequency and became rapidly fixed in parallel evolving experimental populations if they provided a rather minor fitness gain [60]. LSD formation is an example of reductive genome evolution; like IS proliferation, reductive evolution is characteristic for bacteria evolving into host-dependent pathogens [56, 61].

Several genes harbored point mutations in independent (*i*.*e*. patient-specific) ST32 lineages, a characteristic of parallel (convergent) evolution. Strikingly, most of these genes have not been reported to be subject to parallel evolution in *B*. *dolosa*, a distantly related Bcc bacterium whose genetic evolution in chronic CF infection was studied in detail [22, 50]. The handful of shared genes (4/16) either received lowest numbers of parallel mutations in ST32 (*spoT*, *fixL*, *rpoD*) or were mutated due to antibiotic selective pressure (*gyrA*) (Fig 3). Altogether, this implies that evolution of both Bcc species in chronic CF infection is driven by different selective forces. This distinction may be genetically-grounded, predetermining both primarily environmental bacteria to establish persistent infection by own independent means.

### Oxidative stress and transition metals

Genes linked to oxidative stress response and transition metal metabolism were markedly represented among the most mutated genes (Fig 3). These functional categories have not been reported to undergo adaptive within-patient evolution either in *B*. *dolosa* or in the well-characterized CF pulmonary pathogen *Pseudomonas aeruginosa* [62]. Proteins encoded by the three genes are involved in protection against two reactive oxygen species (ROS): hydrogen peroxide (KatG) and hypochlorous acid (YedY, MoeA1). Both ROS are produced by leukocytes as bactericidal agents. Thus, our findings point to a fundamental role of host immune system in driving *B*. *cenocepacia* evolution during chronic CF infection. Chronic pulmonary infections are accompanied by persistent inflammation and neutrophil infiltration. Extracellular hypochlorous acid production by CF neutrophils is not compromised [63] and results in significant chlorination damage in sputum [64]. Although ST32 isolates from our collection which carried various presumably adaptive mutations in YedY and/or MoeA1 were uniformly sensitive to sodium hypochlorite *in vitro*, the unprecedented extent of parallelism suggests principal importance of these mutations *in vivo*. YedY and MoeA1 were frequently mutated not only in 8 patients who developed CS (Fig 3), but also in 12 control non-CS patients (Table 1) and in other *B*. *cenocepacia* IIIA strains (S9 Table [40]). Repair of oxidized periplasmic proteins is thus a previously unrecognized target of adaptive evolution during chronic infection progress in *B*. *cenocepacia*. Other genes carrying parallel mutations revealed transition metal (copper) metabolism as a target of adaptive evolution (see below). Copper has recently been shown to concentrate in macrophage phagolysosomes, aiding in the clearance of ingested bacteria and fungi [65–67]; suggesting possible functional connection between these two categories.

### Is there a link to cepacia syndrome?

Pathogenesis of the fatal CS still remains largely unknown. All ST32 isolates initially characterized by WGS originated from CS patients. In addition, polymorphisms were analyzed in oxidative stress and transition metal metabolism genes in ST32 populations from non-CS patients. Upon the comparison of the two datasets, several genes were detected where mutations segregated among CS and non-CS patients; however, due to inevitably small numbers of patients included, the differences did not reach statistical significance. *copCD* operon was inactivated in every CS patient but one (7/8; 88%) by independent deletions or IS insertions, these events were biased towards late isolates (S2 Fig). In contrast, only in 5/12 (42%) populations from non-CS patients were these mutations present in detectable frequencies (Table 1) (*p* = 0.07, Fisher´s exact test). In the copper-sensing histidine kinase CusS, only mutations from CS patients localized exclusively to the DHp domain (Fig 4, S10 Table). Mutations in copper-related genes did not modulate the measured *in vitro* sensitivity of ST32 towards copper; however, their abundance and/or specific pattern in CS isolates strongly suggest a yet undisclosed role of this metal in CS pathogenesis.

Catalase KatG was mutated in 2/12 non-CS patients (17%). In contrast, isolates from 4/8 CS patients (50%) carried nonsynonymous mutations in *katG* (*p* = 0.16, Fisher´s exact test); another CS patient was colonized with population whose *katG* region was multiplicated (Fig 5). Curiously, some epidemic *B*. *cenocepacia* isolates have been known to possess a paralog of KatG, which is 76% identical and performs different cellular functions than the canonical KatG [35]. Indeed, the hypervirulent ET12 lineage was the only clone among *B*. *cenocepacia* IIIA strains sequenced by Lee *et* al. [40] where *katG* paralog was present in genomic sequences. We speculate that the presence of *katG* mutations might indicate unfavorable outcome of chronic ST32 infection. This is further underlined by a recent fatality case: patient II (Table 1), upon completion of WGS and DPS analyses, underwent lung transplantation and developed CS within several months. A functional link between ROS protection and motility, another CS predictor we have reported previously to segregate between CS and non-CS patients [68], is lacking.

On a final note, we would like to emphasize that our results point to macrophages, the type of professional phagocytes which rely on both hydrogen peroxide and copper for their bactericidal activity, as putative key players behind the development of CS. *B*. *cenocepacia* has been known for its affinity to macrophages; several mechanisms were described which enable intracellular persistence [69] and the importance of macrophages in infection establishment has freshly been demonstrated [70]. Importantly, CF macrophages differ from normal macrophages by exhibiting both hyperinflammatory response to bacteria and their impaired phagocytosis and killing [71]. The observed attenuation of protective mechanisms against antimicrobial agents during within-patient evolution of ST32 is counterintuitive; in *Mycobacterium tuberculosis*, inactivation of *katG* or copper-resistance mechanisms lead to decreased virulence as a result of impaired survival of oxidative burst in macrophages [72, 73]. We hypothesize that under increased stress encountered in CF macrophages, evolved bacteria might activate physiological processes (*e*.*g*. global stress response, persistence) which in turn can modulate the course of intracellular infection. The precise roles of macrophages (at host side) and defense mechanisms (at pathogen side) in chronic infection outcome warrant further investigation.

## Materials and methods

### Bacterial isolates

32 isolates of the *B*. *cenocepacia* epidemic clone (CZ1 [18], multilocus sequence type ST32) were collected during routine microbiological examinations of CF patients at the Centre for Cystic Fibrosis, Motol University Hospital, Prague, and kept deep-frozen. Frozen stocks were streaked and a single colony was selected and directly re-stocked; for all subsequent procedures, aliquots of these final stocks were plated and grown bacterial populations were used directly to minimize introduction of unwanted genetic variability.

### Reference genome analysis

The complete annotated genome of ST32 isolate *B*. *cenocepacia* 1232 (Genbank ID: GCA_001484665.1) was used as reference for all comparative analyses.

#### Whole-genome phylogeny

Phylogeny was reconstructed from complete or draft genomes of *B*. *cenocepacia recA* group IIIA representing various MLST sequence types as deposited at Genbank on March 1^st^, 2017 (S2 Table). Genomic sequences were uploaded to the CSIPhylogeny v1.4 website (https://cge.cbs.dtu.dk/services/CSIPhylogeny/) and automatically processed with default settings. SNP analysis was carried out using a set of algorithms as described in [74] and FastTree [75] was used for phylogram construction. WGS tree was constructed from 95,357 variant nucleotide positions.

#### IS identification

Insertion sequences (IS) were identified *de novo* using the in-house Repeat Finder plugin in Geneious R9 platform (Biomatters Ltd.) with following settings: minimum repeat length 200 bp, maximum 5% mismatches. This resulted in identification of regions of reference genome which were repeated elsewhere in the sequence; both intra- and inter-replicon repeats were identified. Identical or near-identical repeats were grouped together (groups 01 to 13). The encoded proteins were assigned to known IS families using IS Finder [76] (S5 Table).

#### GI prediction

Putative genomic islands (GIs) were detected as follows: GIs specific for ST32 and missing in the well-characterized epidemic strain *B*. *cenocepacia* J2315 (called GiST32), were delineated as continuous DNA regions longer than 10 kb (flanked at both sides by homologous regions) which did not encode homologous proteins, as inferred from orthologs precompiled at www.burkholderia.com [77]. The results were further confirmed with Progressive MAUVE whole genome pairwise alignment [78] (S4 Table). GIs shared with J2315 were identified by BLAST search [79] of nucleotide sequences of GIs previously detected in J2315 genome [10, 52] against the ST32 reference.

### Whole-genome sequencing

Bacteria for genomic DNA preparation were harvested from an agar plate culture (Mueller-Hinton, Oxoid) inoculated directly from frozen stock and grown overnight at 37°C. DNA was isolated using ChargeSwitch gDNA Mini Bacteria Kit (Invitrogen) and quantified using Quant-iT PicoGreen dsDNA Assay Kit (Invitrogen). Sequencing libraries were prepared using Nextera XT DNA Library Preparation Kit (Illumina) and sequenced on the MiSeq platform (Illumina) using MiSeq Reagent Kit v2 (300 cycle) (Illumina), resulting in 2 x 150 bp paired-end reads. Sequencing reads are available from Genbank (Bioproject PRJNA397653).

### Bioinformatic analysis of whole-genome sequencing data

#### Read mapping

Paired-end reads were mapped to the complete reference genome of ST32 isolate 1232 using Geneious R9 platform [80]. The in-house Geneious read mapper [81] was used with following custom mapping settings: max. 10% gaps per read, max. 5% mismatches per read. Sequencing read coverage was higher than 60x for all sequenced genomes (average coverage for chromosome 1).

#### Coverage analysis

Regions of low sequencing read coverage (≤30) were called using the Geneious in-house functionality. Continuous or adjacent low coverage regions were visually inspected and those with zero or negligible coverage and length exceeding 10 kb were collected (S3 Table). Adjacent low coverage regions were merged if separated by repetitive sequences (*e*.*g*. ISs) to reflect false positive coverage of repeated regions introduced during read mapping.

#### Variant calling

Variants with frequency ≥80% and coverage ≥15 were called using the Geneious in-house functionality. SNPs with average quality lower than 25 were discarded. Variants within repeated DNA regions of reference genome (see above) were discarded (S7 Table).

#### SNP analysis

Among variants (selected as described above), SNPs informative of within-patient evolution were extracted as follows: All SNPs which arose before establishment of patient-specific lineages (*i*.*e*. SNPs inherited vertically from common ancestor of multiple lineages) were discarded. SNPs with coverage ≤30 and abnormally clustered SNPs were checked on assemblies of mapped sequencing reads for assembly continuity; false SNPs (*e*.*g*. novel IS insertions, deletions) were discarded. SNP in isolates 3A and 7C which violate the patient-specific clustering were excluded. SNPs passing the criteria are listed in S7 Table.

#### Phylogenetic analysis

Consensus sequences derived from sequencing read mapping to reference genome (as described above) were used for inference of phylogeny of ST32 isolates. Consensus sequences were uploaded to the CSIPhylogeny v1.4 website (https://cge.cbs.dtu.dk/services/CSIPhylogeny/) and automatically processed with default settings. SNP analysis was carried out using a set of algorithms as described in [74] and FastTree [75] was used for phylogram construction. WGS tree was constructed from 2,754 variant nucleotide positions.

#### IS insertions

IS insertions were computationally detected using ISMapper [30]. The software utilizes user-provided IS nucleotide sequences and paired-end sequencing reads to identify and locate IS insertions with respect to reference. Among ISMapper output, calls corresponding to IS insertions already present in reference and novel IS insertions continuous at both ends with reference genomic sequence were retained; the remaining calls were deemed unreliable and discarded [30]. Insertions within repeated DNA regions detected in reference genome (see above) and in their immediate vicinity (≤100 bp) were discarded. Reference genome regions rich in very closely positioned ISs were left unresolved and omitted from analysis (Fig 2). All remaining calls were validated by inspection of called insertion sites in Geneious read mapping assemblies for particular isolates (S6 Table). Positions of IS insertions and deletions in metal-related genes (S2 Fig) were scrutinized visually for all events reported by ISMapper. The first nucleotide position where sequence homogeneity of assembly was violated by clustered mutations was regarded as site of IS insertion or as deletions border (S2 Fig).

### Analysis of ST32 population polymorphism

Total DNA extracted from sputa of 12 CF patients with Amplicor Respiratory Specimen Preparation Kit (Roche) during periodic routine molecular microbiological examination in 2016 was used as template for PCR reactions. Q5 Hot Start High-Fidelity DNA Polymerase (New England Biolabs) was used to minimize amplification errors. Reaction mixtures with the Q5 High GC Enhancer were prepared according to manufacturer´s recommendations in a final volume of 50 μl, containing 1 μl template DNA and 0.5 μM of each primer (S8 Table). PCR reactions were run for 35 cycles at annealing temperature 67°C.

#### Point mutations

PCR reactions were pooled for each patient and amplified DNA was purified with AMPure XP kit (Beckton Dickinson). Libraries were prepared from pooled purified PCR products and sequenced in the same way as with whole-genome sequencing (see above). The sequencing reads were mapped to sequences of respective genes in Geneious, with mapping to structural variants enabled. Variants over 2% frequencies in population were called. SNPs with average quality lower than 25 were discarded.

#### Structural variation

PCR reactions were resolved on agarose gel electrophoresis with ethidium bromide staining. PCR reactions on template DNA purified from WGS strains containing known structural variations (IS insertion, deletion) or intact genes were run in parallel as controls.

### Determination of minimum inhibitory concentration (MIC)

Aliquots of frozen stocks were plated on Mueller-Hinton agar plates (Oxoid) and incubated overnight. Grown bacteria were transferred into 1 ml Luria broth (Sigma) to obtain a suspension with OD_600_ approximately 0.05–0.1. The cultures were incubated at 37°C with shaking for 2 hours to reach mid-exponential phase. Copper(II) chloride, hydrogen peroxide and sodium hypochlorite (Sigma) solutions in Luria broth were freshly prepared at 64 mM, 0.1% (vol/vol) and 0.5% (w/vol) concentrations, respectively, and sterile-filtered. The solutions were serially diluted 2-fold with sterile Luria broth, 100 μl were transferred to MIC microtiter plate wells and inoculated with 1 μl of bacterial cultures. The MIC plates were incubated aerobically for 24 hours at 37°C and MICs were recorded as the minimal concentration of antimicrobial compound which resulted in no visible growth. The experiments were repeated in three biological replicates.

### Ethics statement

Sputum samples were taken with the written informed consent of the CF adult subjects. The study was approved by the Ethics Committee for Multicentric clinical trials of the University Hospital Motol, Prague on September 22, 2010, No.4.2.6.

## Supporting information

S1 Fig Whole-genome phylogeny of *B*. *cenocepacia recA* group IIIA genomes. Each MLST sequence type is represented with one genome (see S2 Table). Complete genomes are denoted in bold. The ST32/ST33 lineage and the epidemic ET12 lineage are indicated. The tree was constructed from 95,357 variant nucleotide positions using the CSIPhylogeny pipeline.
(PDF)

S2 Fig Structural variation of genes involved in metal metabolism. Genes undergoing parallel IS insertions and/or deletions are denoted by gray arrows. Novel IS insertions are marked with dots and their positions, orientations and types are indicated. Deleted regions are crossed out. Deletions and IS insertions were confirmed on assemblies of mapped sequencing reads (see Materials and Methods).
(PDF)

S3 Fig SNP distribution among *B*. *cenocepacia* ST32 genes. The grey columns denote total numbers of genes containing given numbers of nonsynonymous or synonymous SNPs among the ST32 WGS dataset (see Materials and Methods). Identical mutations (*gyrA*, *katG*) were counted separately if they arose independently in patient-specific lineages (as deduced from WGS phylogeny). SNPs specific for hypermutable isolates 2A-2D were excluded from analysis. The best-fit Poisson distribution values (method of least squares) are shown as white columns.
(PDF)

S1 Table Summary information about 8 CS patients from whom the sequenced *B*. *cenocepacia* ST32 were isolated. (XLSX)

S2 Table Whole genome sequences of *B*. *cenocepacia* IIIA used for comparative genomic analyses. (DOCX)

S3 Table LSDs in ST32 genomes sequenced in this study. Only regions of missing coverage larger than 10 kb are reported.
(XLSX)

S4 Table Putative genomic islands in ST32 genome (GiST32). (XLSX)

S5 Table IS incidence in *B*. *cenocepacia* IIIA complete genomes. (XLSX)

S6 Table IS insertions in genomes of ST32 isolates sequenced in this study. (XLSX)

S7 Table Variant calling from mapping of WGS sequencing reads to the reference ST32 isolate 1232. Called variants with frequency ≥80%, coverage ≥15 and average quality ≥25 (only SNPs, not applied to other mutations) are reported. SNPs informative of within-patient evolution (see Materials and Methods) are listed in a separate sheet.
(XLSX)

S8 Table List of primers used for PCR amplification of genes under parallel evolution. (DOCX)

S9 Table Nonsynonymous mutations within selected genes under parallel evolution in various *B*. *cenocepacia* IIIA longitudinal clonal lineages. Only mutations present in a subset of isolates of a particular RAPD type (*i*.*e*. that have arisen after the divergence of RAPD types) are denoted. The numbers in parentheses denote the percent of isolates from a particular patient that carried the mutation. Data from [40].
(DOCX)

S10 Table Alignment of *B*. *cenocepacia* ST32 proteins and homologs with determined crystal structures. The mutated and catalytically important residues are colored as in Fig 4. Functional domains (DHp in CusS, βi4 in RpoB) are denoted with grey shading.
(DOCX)
