# Supplementary figures and images for: What matters in chronic Burkholderia cenocepacia infection in cystic fibrosis: Insights from comparative genomics
Source: PLoS Pathog. 2017 Dec 11;13(12):e1006762. doi: 10.1371/journal.ppat.1006762 (PMC5739508; doi:10.1371/journal.ppat.1006762)

Chromosome 1

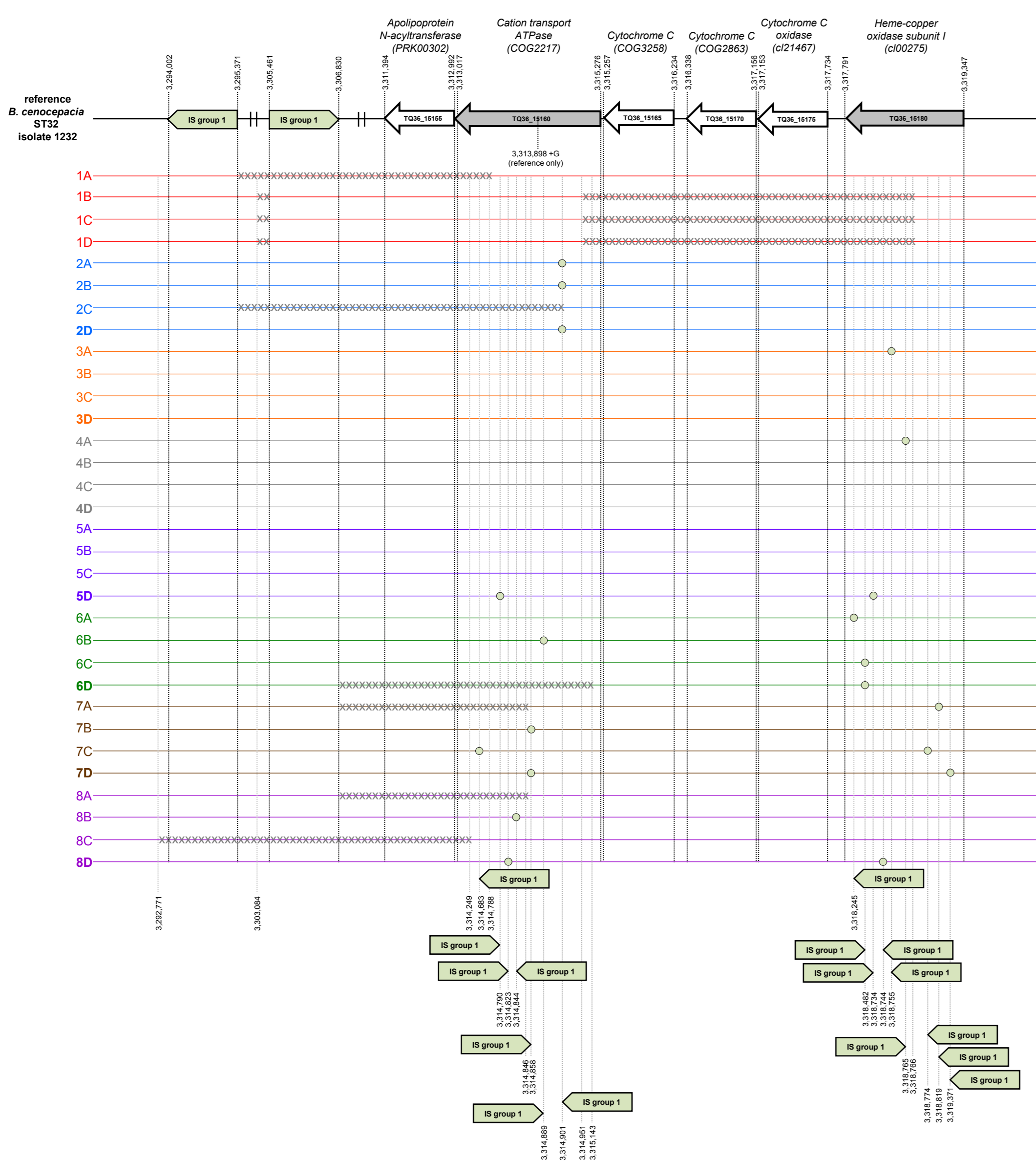

Chromosome 2

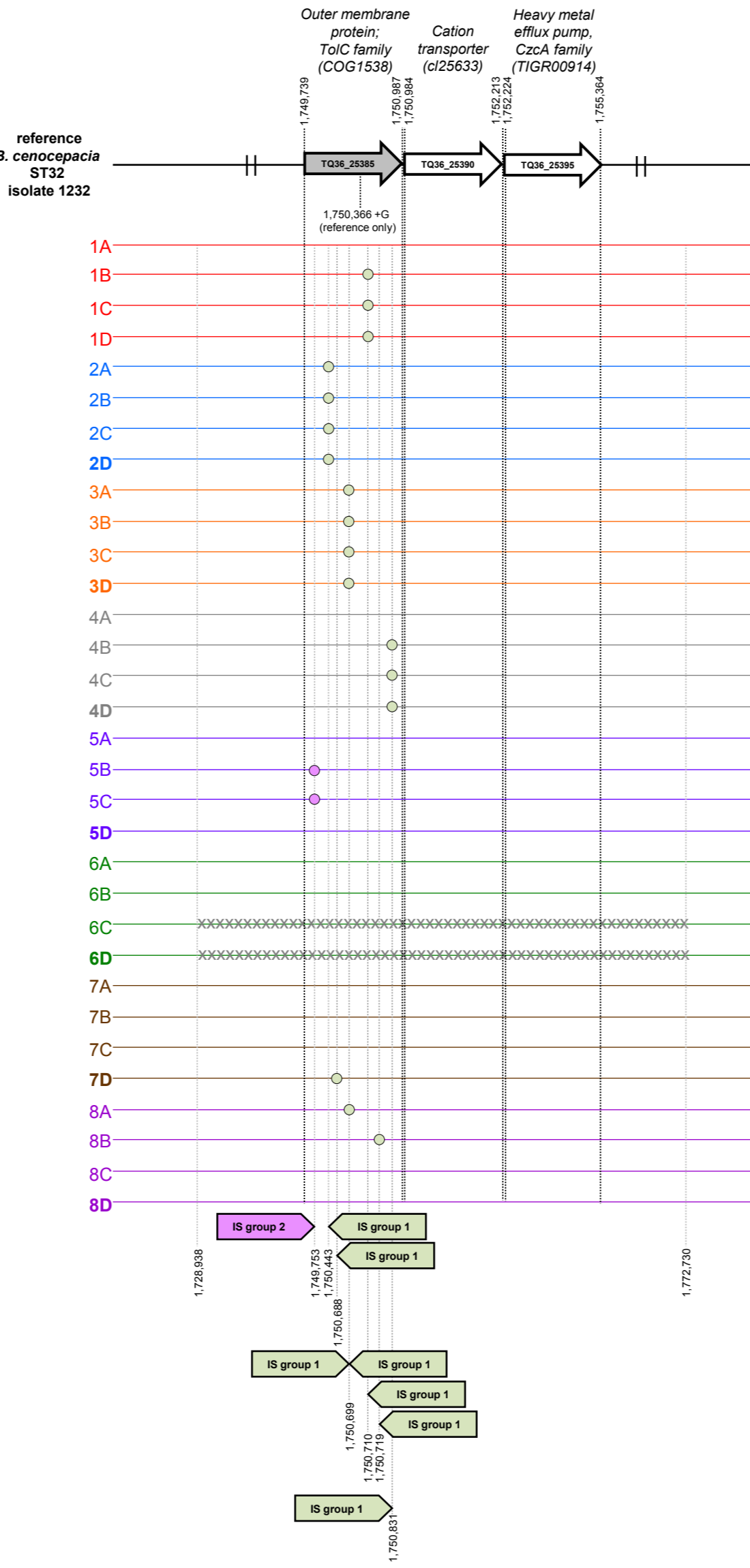

Plasmid

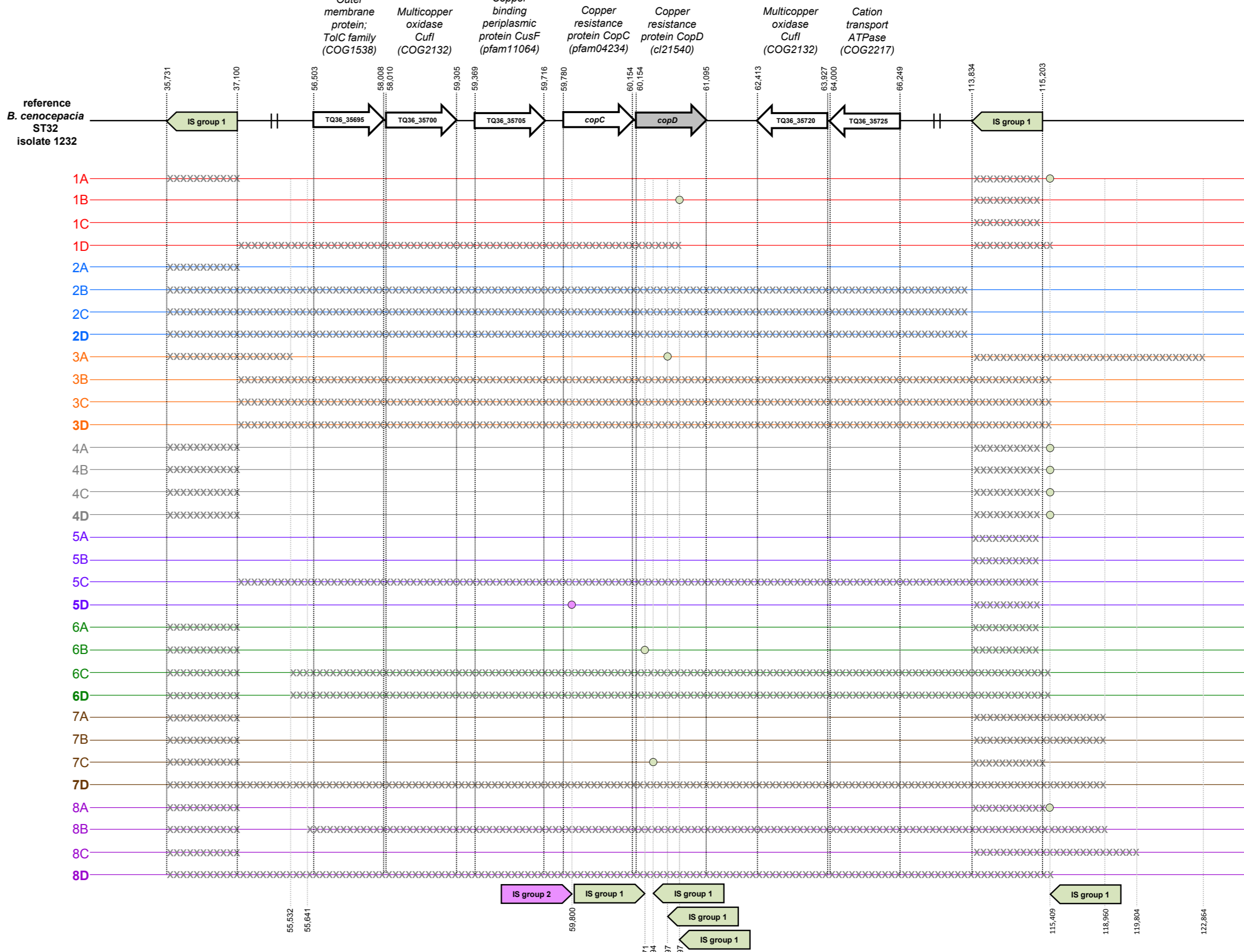

Supplement: S2 Fig — Genes undergoing parallel IS insertions and/or deletions are denoted by gray arrows. Novel IS insertions are marked with dots and their positions, orientations and types are indicated. Deleted regions are crossed out. Deletions and IS insertions were confirmed on assemblies of mapped sequencing reads (see Materials and Methods). (PDF) [file ppat.1006762.s002.pdf]

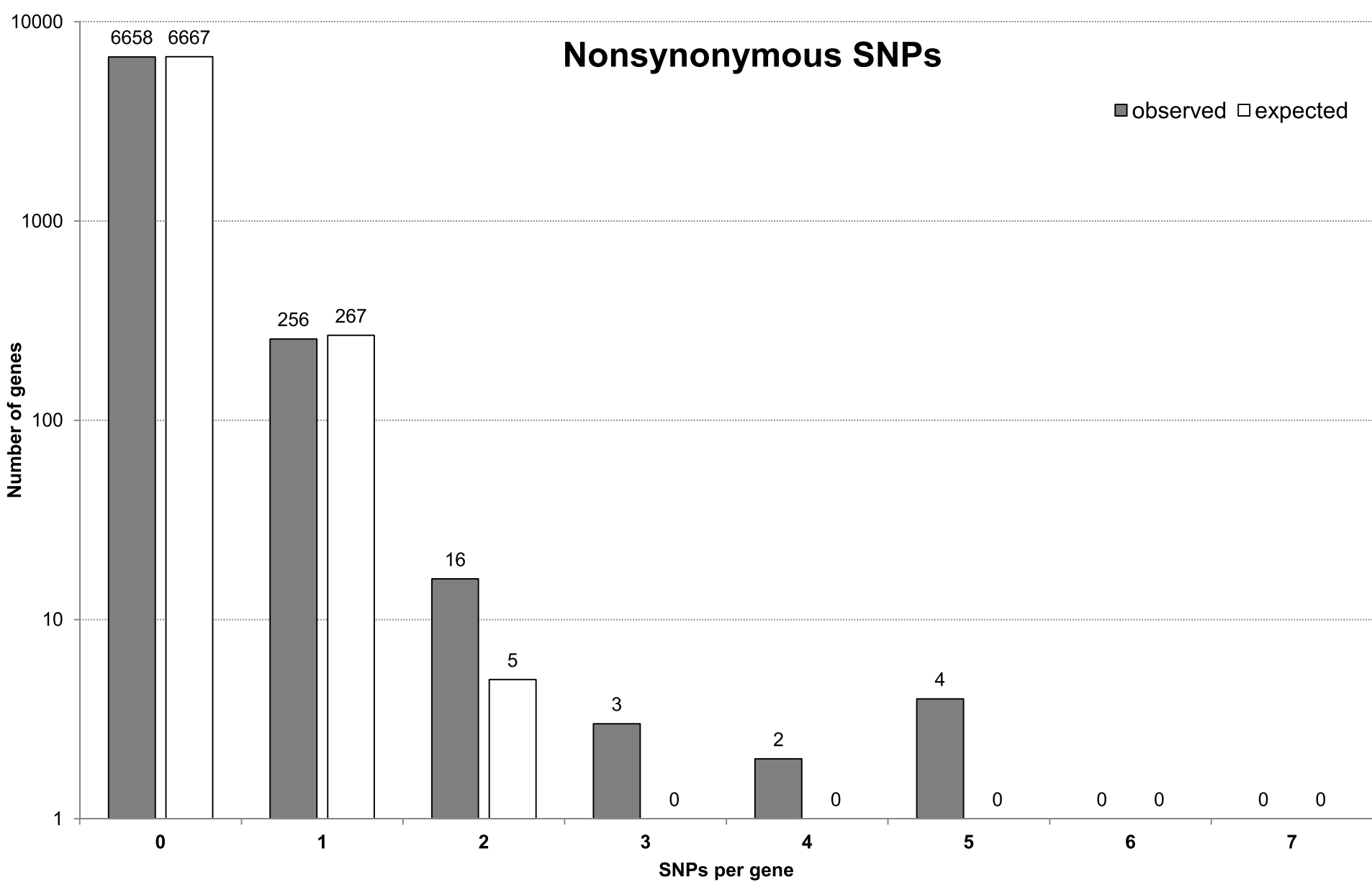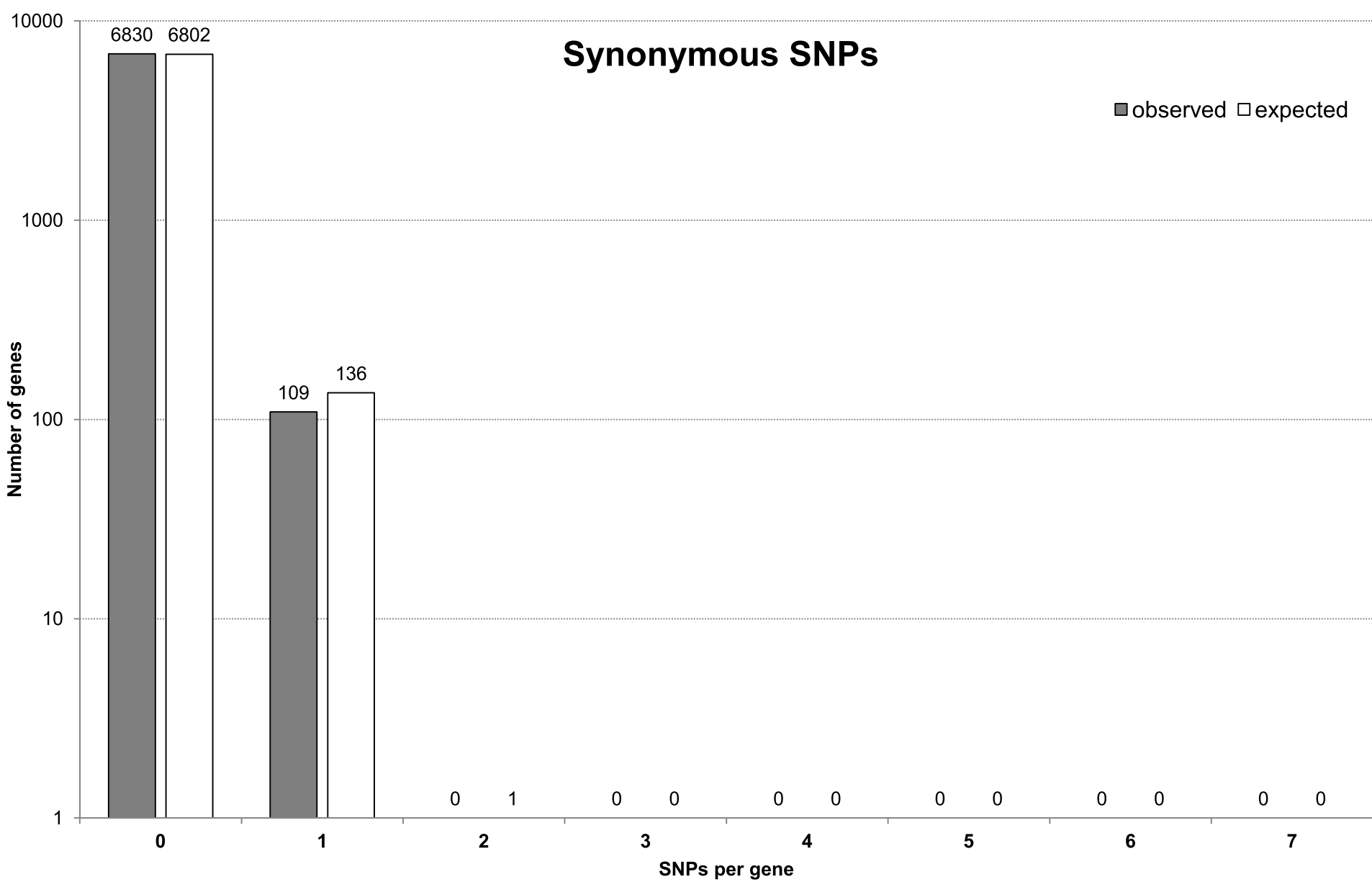

Supplement: S3 Fig — The grey columns denote total numbers of genes containing given numbers of nonsynonymous or synonymous SNPs among the ST32 WGS dataset (see Materials and Methods). Identical mutations (gyrA, katG) were counted separately if they arose independently in patient-specific lineages (as deduced from WGS phylogeny). SNPs specific for hypermutable isolates 2A-2D were excluded from analysis. The best-fit Poisson distribution values (method of least squares) are shown as white columns. (PDF) [file ppat.1006762.s003.pdf]
