# Supplementary material for: What matters in chronic Burkholderia cenocepacia infection in cystic fibrosis: Insights from comparative genomics
Source: PLoS Pathog. 2017 Dec 11;13(12):e1006762. doi: 10.1371/journal.ppat.1006762 (PMC5739508; doi:10.1371/journal.ppat.1006762)
Supplement: S2 Table — (DOCX) [file ppat.1006762.s005.docx]

**S2 Table. Whole genome sequences of *B. cenocepacia* IIIA used for comparative genomic analyses.**

| **Strain** | **Isolation Year** | **Isolation Country** | **Isolation Source** | **MLST** | **Status** | **Accession** | **Reference** |
| --- | --- | --- | --- | --- | --- | --- | --- |
| [*Burkholderia cenocepacia*](https://www.ncbi.nlm.nih.gov/genome/475?genome_assembly_id=260721) 842 | 2014 | Malaysia | nonCF clinical | 1298 | complete | [GCA_001606115.1](https://www.ncbi.nlm.nih.gov/assembly/GCA_001606115.1) | x |
| [*Burkholderia cenocepacia*](https://www.ncbi.nlm.nih.gov/genome/475?genome_assembly_id=260721) 895 | 2014 | Malaysia | nonCF clinical | 628 | complete | [GCA_001606135.1](https://www.ncbi.nlm.nih.gov/assembly/GCA_001606135.1) | x |
| [*Burkholderia cenocepacia* H111](https://www.ncbi.nlm.nih.gov/genome/475?genome_assembly_id=229618) | 1993 | Germany | CF sputum | 220 | complete | [GCA_000236215.4](https://www.ncbi.nlm.nih.gov/assembly/GCA_000236215.4) | [1] |
| [*Burkholderia cenocepacia* J2315](https://www.ncbi.nlm.nih.gov/genome/475?genome_assembly_id=300159) | 1989 | UK | CF sputum | 28 | complete | [GCA_000009485.1](https://www.ncbi.nlm.nih.gov/assembly/GCA_000009485.1) | [2] |
| [*Burkholderia cenocepacia*](https://www.ncbi.nlm.nih.gov/genome/475?genome_assembly_id=260721) [MSMB384WGS](https://www.ncbi.nlm.nih.gov/genome/475?genome_assembly_id=281623) | 2008 | Australia | water | 1300 | complete | [GCA_001718895.1](https://www.ncbi.nlm.nih.gov/assembly/GCA_001718895.1) | [3] |
| [*Burkholderia cenocepacia*](https://www.ncbi.nlm.nih.gov/genome/475?genome_assembly_id=260721) ST32 | 1997 | Czech Republic | CF sputum | 32 | complete | [GCA_001484665.1](https://www.ncbi.nlm.nih.gov/assembly/GCA_001484665.1) | x |
| [*Burkholderia cenocepacia*](https://www.ncbi.nlm.nih.gov/genome/475?genome_assembly_id=260721) [VC12308](https://www.ncbi.nlm.nih.gov/genome/475?genome_assembly_id=307158) | 2004 | Canada | CF sputum | 306 | complete | [GCA_001999885.1](https://www.ncbi.nlm.nih.gov/assembly/GCA_001999885.1) | [4] |
| [*Burkholderia cenocepacia*](https://www.ncbi.nlm.nih.gov/genome/475?genome_assembly_id=260721) VC1254 | 1985 | Canada | CF sputum | 32 | complete | [GCA_001999925.1](https://www.ncbi.nlm.nih.gov/assembly/GCA_001999925.1) | [4] |
| [*Burkholderia cenocepacia*](https://www.ncbi.nlm.nih.gov/genome/475?genome_assembly_id=260721) [VC2307](https://www.ncbi.nlm.nih.gov/genome/475?genome_assembly_id=307158) | 1987 | Canada | CF sputum | 210 | complete | [GCA_001999805.1](https://www.ncbi.nlm.nih.gov/assembly/GCA_001999805.1) | [4] |
| [*Burkholderia cenocepacia*](https://www.ncbi.nlm.nih.gov/genome/475?genome_assembly_id=260721) 34_BCEN | 2012 | USA | nonCF clinical | 242 | draft | [GCA_001056435.1](https://www.ncbi.nlm.nih.gov/assembly/GCA_001056435.1) | [5] |
| [*Burkholderia cenocepacia*](https://www.ncbi.nlm.nih.gov/genome/475?genome_assembly_id=260721) [AE_MEH_16](https://www.ncbi.nlm.nih.gov/genome/475?genome_assembly_id=281805) | 2015 | Lebanon | clinical | 602 | draft | [GCA_001720525.1](https://www.ncbi.nlm.nih.gov/assembly/GCA_001720525.1) | x |
| [*Burkholderia cenocepacia*](https://www.ncbi.nlm.nih.gov/genome/475?genome_assembly_id=260721) [FL-6-2-30-S1-D2](https://www.ncbi.nlm.nih.gov/genome/475?genome_assembly_id=262802) | 2012 | USA | soil | 964 | draft | [GCA_001524105.1](https://www.ncbi.nlm.nih.gov/assembly/GCA_001524105.1) | [3] |
| [*Burkholderia cenocepacia*](https://www.ncbi.nlm.nih.gov/genome/475?genome_assembly_id=260721) K56-2 | 1999 | Canada | CF sputum | 30 | draft | [GCA_000981305.1](https://www.ncbi.nlm.nih.gov/assembly/GCA_000981305.1) | x |
| [*Burkholderia cenocepacia*](https://www.ncbi.nlm.nih.gov/genome/475?genome_assembly_id=260721) [MSMB364WGS](https://www.ncbi.nlm.nih.gov/genome/475?genome_assembly_id=262803) | 2008 | Australia | water | 1299 | draft | [GCA_001531965.1](https://www.ncbi.nlm.nih.gov/assembly/GCA_001531965.1) | [3] |
| [*Burkholderia cenocepacia*](https://www.ncbi.nlm.nih.gov/genome/475?genome_assembly_id=260721) VC3917 | 1990 | Canada | CF sputum | 33 | draft | [GCA_001984355.1](https://www.ncbi.nlm.nih.gov/assembly/GCA_001984355.1) | [4] |
| [*Burkholderia cenocepacia*](https://www.ncbi.nlm.nih.gov/genome/475?genome_assembly_id=260721) VC6916 | 1995 | Canada | CF sputum | 278 | draft | [GCA_001993215.1](https://www.ncbi.nlm.nih.gov/assembly/GCA_001993215.1) | [4] |
| [*Burkholderia cenocepacia*](https://www.ncbi.nlm.nih.gov/genome/475?genome_assembly_id=260721) VC7292 | 1995 | Canada | CF sputum | 234 | draft | [GCA_001993435.1](https://www.ncbi.nlm.nih.gov/assembly/GCA_001993435.1) | [4] |
| [*Burkholderia cepacia*](https://www.ncbi.nlm.nih.gov/genome/475?genome_assembly_id=260721) [DDS 7H-2](https://www.ncbi.nlm.nih.gov/genome/10703?genome_assembly_id=208817) | 2005 | Australia | aerosol | 807 | draft | [GCA_000755805.1](https://www.ncbi.nlm.nih.gov/assembly/GCA_000755805.1) | [6] |

1. Carlier A, Agnoli K, Pessi G, Suppiger A, Jenul C, Schmid N, et al. Genome sequence of *Burkholderia cenocepacia* H111, a cystic fibrosis airway isolate. Genome Announc. 2014;2(2). Epub 2014/04/10. doi: 10.1128/genomeA.00298-14. PubMed PMID: 24723723; PubMed Central PMCID: PMCPMC3983312.

2. Holden MT, Seth-Smith HM, Crossman LC, Sebaihia M, Bentley SD, Cerdeño-Tárraga AM, et al. The genome of *Burkholderia cenocepacia* J2315, an epidemic pathogen of cystic fibrosis patients. J Bacteriol. 2009;191(1):261-77. doi: 10.1128/JB.01230-08. PubMed PMID: 18931103; PubMed Central PMCID: PMCPMC2612433.

3. Sahl JW, Vazquez AJ, Hall CM, Busch JD, Tuanyok A, Mayo M, et al. The effects of signal erosion and core genome reduction on the identification of diagnostic markers. MBio. 2016;7(5). Epub 2016/09/20. doi: 10.1128/mBio.00846-16. PubMed PMID: 27651357; PubMed Central PMCID: PMCPMC5030356.

4. Lee AH, Flibotte S, Sinha S, Paiero A, Ehrlich RL, Balashov S, et al. Phenotypic diversity and genotypic flexibility of *Burkholderia cenocepacia* during long-term chronic infection of cystic fibrosis lungs. Genome Res. 2017;27(4):650-62. Epub 2017/03/21. doi: 10.1101/gr.213363.116. PubMed PMID: 28325850; PubMed Central PMCID: PMCPMC5378182.

5. Roach DJ, Burton JN, Lee C, Stackhouse B, Butler-Wu SM, Cookson BT, et al. A year of infection in the intensive care unit: Prospective whole genome sequencing of bacterial clinical isolates reveals cryptic transmissions and novel microbiota. PLoS Genet. 2015;11(7):e1005413. Epub 2015/07/31. doi: 10.1371/journal.pgen.1005413. PubMed PMID: 26230489; PubMed Central PMCID: PMCPMC4521703.

6. Daligault HE, Davenport KW, Minogue TD, Bishop-Lilly KA, Broomall SM, Bruce DC, et al. Whole-genome assemblies of 56 burkholderia species. Genome Announc. 2014;2(6). Epub 2014/11/20. doi: 10.1128/genomeA.01106-14. PubMed PMID: 25414490; PubMed Central PMCID: PMCPMC4239345.
