# Supplementary material for: What matters in chronic Burkholderia cenocepacia infection in cystic fibrosis: Insights from comparative genomics
Source: PLoS Pathog. 2017 Dec 11;13(12):e1006762. doi: 10.1371/journal.ppat.1006762 (PMC5739508; doi:10.1371/journal.ppat.1006762)
Supplement: S8 Table — (DOCX) [file ppat.1006762.s011.docx]

**S8 Table. List of primers used for PCR amplification of genes under parallel evolution.**

| **Primer name** | **Sequence (5´→3´)** | **Position in Reference** | **Replicon** | **Amplicon Length** |
| --- | --- | --- | --- | --- |
| YedY_F | TCTTTTGTAATGGGCGGGCG | 3,749,959 - 3,749,978 | ch1 | 1,246 |
| YedY_R | GCGAACACCAGCACCTTGG | 3,751,204 - 3,751,186 |  |  |
| MoeA_F | CGACGAGCGTGCGTTTTACAATG | 3,251,321 - 3,251,299 | ch1 | 1,668 |
| MoeA_R | CGCTCAGGATCGTGCTCAGT | 3,249,658 - 3,249,677 |  |  |
| CusS_F | ACTCGTGTGGGACGTGAACT | 1,891,918 - 1,891,899 | ch2 | 1,765 |
| CusR_R | CCAGCATGCTCATCAGGTGT | 1,890,154 - 1,890,173 |  |  |
| RpoB_F | GGCCAACCACCAAGCTTCTC | 3,710,767 - 3,710,786 | ch1 | 4,382 |
| RpoB_R | CTCCGGCTTCTTCACTTCGC | 3,715,148 - 3,715,129 |  |  |
| KatG_F | GCGCACGACATCCCGTA | 3,480,405 - 3,480,421 | ch1 | 2,442 |
| KatG_R | CGCATGTTGAGCATCATTTGCG | 3,482,846 - 3,482,825 |  |  |
| BCAL0155_F | ACGTTCGTTTAGGTTGCCCG | 2,808,741 - 2,808,722 | ch1 | 1,587 |
| BCAL0155_R | CGGACATCAAGGCCTACGAC | 2,807,155 - 2,807,174 |  |  |
|  |  |  |  |  |
| TQ36_15160_F | CTCACAGCCTGAGCATTCCA | 3,312,988 - 3,313,007 | ch1 | 2,315 |
| TQ36_15160_R | CGGAAAACTGCTTGGGGTTG | 3,315,283 - 3,315,302 |  |  |
| TQ36_15180_F | GCAAGCCCTGATGGAACACT | 3,319,414 - 3,319,433 | ch1 | 1,834 |
| TQ36_15180_R | CTGCTGGCTGTGACAGTAGA | 3,317,600 - 3,317,619 |  |  |
| TQ36_25385_F | ATCTGTTGTCGTCGTGAGGG | 1,749,714 - 1,749,733 | ch2 | 1,355 |
| TQ36_25385_R | TGGCCTCCTCTTTGGGTTTG | 1,751,049 - 1,751,068 |  |  |
| CopCD_F | CTGACCGTGGTGAAACTCCA | 59,687 - 59,706 | plasmid | 1,436 |
| CopCD_R | CGTGGACGGACGATAAAGGT | 61,103 - 61,122 |  |  |
