# Supplementary material for: What matters in chronic Burkholderia cenocepacia infection in cystic fibrosis: Insights from comparative genomics
Source: PLoS Pathog. 2017 Dec 11;13(12):e1006762. doi: 10.1371/journal.ppat.1006762 (PMC5739508; doi:10.1371/journal.ppat.1006762)
Supplement: S9 Table — Only mutations present in a subset of isolates of a particular RAPD type (i.e. that have arisen after the divergence of RAPD types) are denoted. The numbers in parentheses denote the percent of isolates from a particular patient that carried the mutation. Data from [40]. (DOCX) [file ppat.1006762.s012.docx]

**S9 Table. Variance in longitudinal isolate series of various *B. cenocepacia* IIIA clonal lineages.**

| **Patient ID** | **ST** | **RAPD Type** | **#**  **Isolates** |  | **BCAL0155** | **MoeA** | **RpoB** | **CusS** | **KatG** | **YedY** |  |
| --- | --- | --- | --- | --- | --- | --- | --- | --- | --- | --- | --- |
| P13 | 32 | 01 | 7 |  | disruption (57) | no change | no change | no change | no change | no change |  |
| P16 | 33 | 01 | 23 |  | no change | no change | no change | 28L→M (30) | no change | no change |  |
| P04 | 28 | 02 | 16 |  | del (50) 81Y→C (50) | no change | 1319V→A (6) | 204R→W (100) | 188W→R (6) | 22T→A (100) |  |
| P11 | 28 | 02 | 8 |  | 270H→R (100) | 156A→T (25) 166A→V (25)  del (100) | 462V→A (38) | 232Y→C (100) | no change | 121E→K (100) |  |
| P12 | 28 | 02 | 8 |  | no change | del (100) | 149V→A (13) | 232Y→C (100) | no change | 121E→K (100) |  |
| P14 | 28 | 02 | 7 |  | no change | 71G→S (14) 174R→G (100) | 352Y→H (14) 839I→T (86)  511F→L (100) | 466V→I (14) | 229D→G (14) | no change |  |
| P15 | 28 | 02 | 7 |  | no change | insI (100) | no change | 427A→S (100) | no change | 22T→M (100) |  |
| P02 | 234 | 04 | 17 |  | no change | 386S→P (12) 116T→I (18) | no change | no change | 570A→E (6) | 24R→C (6)  131D→N (6)  154M→V (12) |  |
| P03 | 234 | 04 | 16 |  | no change | 313G→D (44) | 491E→G (6) | no change | no change | 131D→N (56) |  |
| P05 | 278 | 04 | 14 |  | 270H→R (100) | 415L→P (100) | no change | no change | no change | 165E→G (100) |  |
| P07 | 234 | 04 | 10 |  | no change | no change | no change | no change | no change | no change |  |
| P01 | 210 | 06 | 22 |  | no change | 313G→C (9) | no change | gene lost (91) | no change | 327L→P (5) |  |
| P06 | 210 | 06 | 9 |  | no change | insII (100) | 903P→A (100) | gene lost (100) | no change | no change |  |
| P10 | 210 | 06 | 9 |  | no change | insII (100) | 903P→A (100) | gene lost (100) | no change | 16I→F (11)  151G→S (44) |  |
|  |  |  |  |  |  |  |  |  |  |  |  |
| # nonsynonymous mutations in all patients | | | |  | **4** | **12** | **7** | **6** | **3** | **10** |  |
|  | | | |  |  |  |  |  |  |  |  |
| # patients with nonsynonymous mutations in over 50% isolates | | | |  | **4** | **7** | **3** | **7** | **0** | **7** |  |
|  |  |  |  |  |  |  |  |  |  |  |  |

*Only mutations that have arisen after the divergence of RAPD types are denoted. The numbers in parentheses denote the percent of isolates from a particular patient that carried the mutation. Data from Lee et al., Genome Res 2017 [1]*

1. Lee AH, Flibotte S, Sinha S, Paiero A, Ehrlich RL, Balashov S, et al. Phenotypic diversity and genotypic flexibility of *Burkholderia cenocepacia* during long-term chronic infection of cystic fibrosis lungs. Genome Res. 2017;27(4):650-62. Epub 2017/03/21. doi: 10.1101/gr.213363.116. PubMed PMID: 28325850; PubMed Central PMCID: PMCPMC5378182.
