# Supplementary material for: What matters in chronic Burkholderia cenocepacia infection in cystic fibrosis: Insights from comparative genomics
Source: PLoS Pathog. 2017 Dec 11;13(12):e1006762. doi: 10.1371/journal.ppat.1006762 (PMC5739508; doi:10.1371/journal.ppat.1006762)
Supplement: S10 Table — The mutated and catalytically important residues are colored as in Fig 4. Functional domains (DHp in CusS, βi4 in RpoB) are denoted with grey shading. (DOCX) [file ppat.1006762.s013.docx]

**S10 Table. Alignment of *B. cenocepacia* ST32 proteins and homologs with determined crystal structures.**

The mutated and catalytically important residues are colored as in Fig 4. Functional domains (DHp in CusS, βi4 in RpoB) are denoted with grey shading.

**CusS**

3DGE:A|PDBID|CHAIN|SEQUENCE ------------------------------------------------------------ 0

ST32 -----MKRSII--LRLSAMFGIVS---LLVFTLVGC-GLFVMMERQLFAELRATIDTRAK 49

3DGE:A|PDBID|CHAIN|SEQUENCE ------------------------------------------------------------ 0

ST32 VAQMIVSHATTAARGRLMQ---------EKLADLEPPDGSTHYQVISDNPAFRFGSPVDG 100

3DGE:A|PDBID|CHAIN|SEQUENCE ------------------------------------------------------------ 0

ST32 VPLDPPFGAFQRYQLND------SSYAVMTKTITLAGAGE-------------------- 134

3DGE:A|PDBID|CHAIN|SEQUENCE ------------------------------------------------------------ 0

ST32 -RPTLQLVVATSCERTQRMLR---RFGWTLAALIATATVITLLL---SR--AVARFGLAP 185

3DGE:A|PDBID|CHAIN|SEQUENCE ----------------------------VEN--VTESKELER-LKRI**D**RMKTEFI**AN**IS**H** 29

ST32 LDRLSQDAASVSATNRRQRLHTDALPPELRDLATSFNGALER-IQQT**Y**ARLEAFN**AD**VA**H** 244

: : . *** . * * ::*

3DGE:A|PDBID|CHAIN|SEQUENCE ELRTPLTAIKAYAETIYNSLGELDLSTLKEFLEVII**D**QSNHLE**NL**L**N**E**L**LDFSRLE**R**KSL 89

ST32 ELRTPISILIGQTQVALTSRD-RSVDRMRQTLQSNL**E**EFGRLR**VI**I**N**D**M**LFLSRSD**R**GER 303

*****:: : .* . . . :.. * :: :* ::*::* ** *

3DGE:A|PDBID|CHAIN|SEQUENCE QINREKVDLCDLVESAVNAIKEFAS**S**HNVNVLF**E**SNVPCPVEAYIDPTRIRQVL**L**NL**L**NN 149

ST32 ATDLKDVSLADEVRRMLDFLEIPLD**E**ARLRAEL**H**G---D-ARAAVDPSLFRRAM**T**NL**L**IN 359

: *.* : : :: . : : .* ** :*:.: *** *

3DGE:A|PDBID|CHAIN|SEQUENCE GVKYSKKDAPDKYVKVILDEKDG**G**VLIIVEDNGIGIPDHAKDRIFEQFYRVDSSLTYEVP 209

ST32 AIQHSAP---GATLNVTITRRDT**L**VEMAVSNPGEPIDPVLRSHVFERFYRLEEARANSKE 416

.:. * . : * * : * : * * ::** *** .

3DGE:A|PDBID|CHAIN|SEQUENCE GTGLGLAITK**E**IVE-**L**H**G**GRIWVESEVGKGSRFFVWIPKDRAGE-DNRQDN--------- 258

ST32 NHGLGLSIVK**A**VAE-**M**H**G**GGVFVACSGG-VNTFGFSVSTQPCSGGPLRPADVAGAVDSAD 474

. ****:* * : * *** : * * . : *

3DGE:A|PDBID|CHAIN|SEQUENCE -------------------- 258

ST32 SANPSGPRDARPAHAPRALH 494

**KatG**

5SYK:A|PDBID|CHAIN|SEQUENCE MSNEAKCPFHQAAGNGTSNRDWWPNQLDLSILHRHSSLSDPMGKDFNYAQAFEKLDLAAV 60

ST32 MSNETKCPFNHTAGSGTTNKDWWPNQLNLNVLHRHSALSDPMDPDFDYAEAFKKLDLAAV 60

****:****:::**.**:*:*******:*.:*****:*****. **:**:**:*******

5SYK:A|PDBID|CHAIN|SEQUENCE KRDLHALMTTSQDWWPADFGHYGGLFIRMA**X**HSAGTYRTADGRGGAGEGQQRFAPLNSWP 120

ST32 KQDLHALMTASQDWWPADFGHYGGLFVRMA**W**HSAGTYRTADGRGGAGGGQQRFAPLNSWP 120

*:*******:****************:*** **************** ************

5SYK:A|PDBID|CHAIN|SEQUENCE DNANLDKARRLLWPIKQKYGRAISWADLLILTGNVALESMGFKTFGFAGGRADTWEPEDV 180

ST32 DNVSLDKARRLLWPIKQKYGRNISWADLLILTGNVALESMGFKTFGYAGGRVDTWEPDDV 180

**..***************** ************************:****.*****:**

5SYK:A|PDBID|CHAIN|SEQUENCE YWGSEKI**WL**ELSGGPNSRYSGDRQLENPLAAVQMGLI**Y**VNPEGPDGNP**D**PVAAARDIRDT 240

ST32 YWGSEKI**WL**ELSGGPNSRYTGKRELESPLAAVQMGLI**Y**VNPEGPDGNP**D**PVAAAHDIRET 240

*******************:*.*:**.***************************:***:*

5SYK:A|PDBID|CHAIN|SEQUENCE FAR**MAM**NDEETVALIAGGHTFGKTHGAGPASNVGAEPEAAGIEAQGLGWKSAYRTGKGAD 300

ST32 FAR**MAM**NDEETVALIAGGHTFGKTHGAGPASNVGPEPEAAGLEEQGLGWKSTFGTGKGKD 300

********************************** ******:* *******:: **** *

5SYK:A|PDBID|CHAIN|SEQUENCE AITSGLEVTWTTTPTQWSHNFFENLFGYEWELTKSPAGAHQWVAKGADAVIPDAFDPSKK 360

ST32 TITSGLEVTWTSTPTKWSNDFFKHLFSYEWELTKSPAGAHQWVAKDADEVIPDAYDASKK 360

:**********:***:**::**::**.******************.** *****:* ***

5SYK:A|PDBID|CHAIN|SEQUENCE HRPTMLTTDLSLRFDPAYEKISRRFHENPEQFADAFARAWFKLTH**R**DMGPRARYLGPEVP 420

ST32 HRPTMLTTDLSLRFDPAYEKISRRFYENPAEFADAFARAWFKLTH**R**DMGPRSRYLGPEVP 420

*************************:*** :********************:********

5SYK:A|PDBID|CHAIN|SEQUENCE AEVLLWQDPIPAVDHPLIDAADAAELKAKVLASGLTVSQLVSTAWAAASTFRGSDKRGGA 480

ST32 AEHLLWQDPIPAVDHPLIDDADVAALKAKVLATGLSVSQLVSTAWASAATFRGSDKRGGA 480

** **************** **.* *******:**:**********:*:***********

5SYK:A|PDBID|CHAIN|SEQUENCE NGARIRLAPQKDWEANQPEQLAAVLETLEAIRTAFNGAQRGGKQVSLADLIVLAGCAGVE 540

ST32 NGARIRLAPQKDWEVNQPAALAAVLEALEGVQKAFNDAQTGGKKVSLADLIVLAGAAGVE 540

**************.*** ******:**.::.***.** ***:***********.****

5SYK:A|PDBID|CHAIN|SEQUENCE QAAKNAGHAVTVPFAPGRADASQEQTDVE**S**MAVLEPVADGFRNYLKGKYRVPAEVLLVDK 600

ST32 QAAKNAGIAITVPFAPGRMDASQEETDVD**A**MAVLEPLADGFRNYLKHAYKTPAEALLVDK 600

******* *:******** *****:***::******:********* *:.***.*****

5SYK:A|PDBID|CHAIN|SEQUENCE AQLLTLSAPEMTVLLGGLRVLGANVGQSRHGVFTAREQALTNDFFVNLLDMGTEWKPTAA 660

ST32 AQLLTLTAPEMTVLVGGLRVLGANVGDSKHGVFTDRPGTLSNDFFANLLDMRTEWKPVSA 660

******:*******:***********:*:***** * :*:****.***** *****.:*

5SYK:A|PDBID|CHAIN|SEQUENCE DADVFEGRDRATGELKWTGTRVDLVFGSHSQLRALAEVYGSADAQEKFVRDFVAVWNKVM 720

ST32 ANDVFEGRDRATGAVKWTGTRVDLIFGSHSQLRALAEVYGSADAQEKFVRDFVAAWNKVM 720

*********** :*********:*****************************.*****

5SYK:A|PDBID|CHAIN|SEQUENCE NLDRFDLA 728

ST32 NLDRFDLA 728

********

**RpoB**

4YG2:C|PDBID|CHAIN|SEQUENCE MVYSYTEKKRIRKDFGKRPQVLDVPYLLSIQLDSFQKFIEQDPE----GQYGLEAAFRSV 56

ST32 MQYSFTEKKRIRKSFAKRPIVHQVPFLLATQLESFSTFLQADVPATQRKPEGLQAAFTSV 60

* **:********.*.*** * :**:**: **:**..*:: * **:*** **

4YG2:C|PDBID|CHAIN|SEQUENCE FPIQSYSGNSELQYVSYRLGEPVFDVQECQIRGVTYSAPLRVKLRLVIYEREAPEG-TVK 115

ST32 FPIVSHNGFARLEFVSYALSSPAFNIKECQQRGLTYCSALRAKVRLVILDKESPNKPVVK 120

*** *:.* :.*::*** *..*.*:::*** **:**.: **.*:**** ::*:*: .**

4YG2:C|PDBID|CHAIN|SEQUENCE DIKEQEVY**M**GEIPLMTDNGTFVINGTER**V**IVSQLHRSPGVFFDSDKGKTHSSGK**V**L**Y**NAR 175

ST32 EVKEQEVY**M**GEIPLMTPTGSFVINGTER**V**IVSQLHRSPGVFFEHDKGKTHSSGK**L**L**F**SAR 180

::************** .*:**********************: **********:*:.**

4YG2:C|PDBID|CHAIN|SEQUENCE IIPYRGSWLDFEFDPKDNLFVR**I**DRRRKLPATIILRALNYTTEQILDLFFEKVIFEIRDN 235

ST32 IIPYRGSWLDFEFDPKGILYFR**V**DRRRKMPVTILLKAIGLTPEQILANFFVFDNFTLMDE 240

****************. *:.*:*****:*.**:*:*:. * **** ** * : *:

4YG2:C|PDBID|CHAIN|SEQUENCE KLQMELVPERL**R**G**E**TASFDIE-ANGKVYVEKG**R**RITARHIRQLEKDDVKLIEVPVEYIAG 294

ST32 GAQLEFVPERL**R**G**E**VARFDITDRDGKVIVQKD**K**RINAKHIRDLEAAKTKFISVPEDYLLG 300

*:*:********.* *** :*** *:*.:**.*:***:** ..*:*.** :*: *

4YG2:C|PDBID|CHAIN|SEQUENCE KVVAKDYIDESTGELICAANMELSLDLLAKLSQSGHKRIE**T**L**F**T**N**DL**D**HG**PY**ISETLRVD 354

ST32 RVLAKNVVDGDTGEVIASANDEVTESVLEKLREAGIKDIQ**T**L**Y**T**N**DL**D**QG**PY**ISSTLRVD 360

:*:**: :* .***:*.:** *:: .:* ** ::* * *:**:*****:*****.*****

4YG2:C|PDBID|CHAIN|SEQUENCE PTNDRLSALVEIYRMMRPGEPPTREAAESLFENLFFS**E**DRYDLSAVGR**M**KFNRSLLREEI 414

ST32 ETTDKTAARIAIYRMMRPGEPPTEEAVEALFNRLFYS**E**EAYDLSKVGR**M**KFNRRVGRDEI 420

*.*: :* : *:**********.**.*:**:.**:**: **** ******** : *:**

4YG2:C|PDBID|CHAIN|SEQUENCE EGSGILSKDDIIDVMKKLIDIRNGKGEVDDIDHL**G**NRRIRS**V**G**E**MAENQFRVGLVRVERA 474

ST32 VGPMTLQDDDILATIKILVELRNGKGEVDDIDHL**G**NRRVRC**V**G**G**LAENQFRAGLVRVERA 480

* *..***: .:* *:::*****************:*.** :******.********

4YG2:C|PDBID|CHAIN|SEQUENCE VKERLSLGDL**D**TLMPQDMINAKPISAAVKE**F**FGSSQLSQFMDQNNPLSEITHKRRISALG 534

ST32 VKERLGQAES**E**NLMPHDLINSKPISSAIRE**F**FGSSQLSQFMDQTNPLSEITHKRRVSALG 540

*****. .: :.***:*:**:****:*::**************.***********:****

4YG2:C|PDBID|CHAIN|SEQUENCE PGGLTRERAGFEVRDVHPTHYGRVCPIETPEGPNIGLINSLSVYAQTNEYGFLETPYRKV 594

ST32 PGGLTRERAGFEVRDVHPTHYGRVCPIETPEGPNIGLINSLALYAHLNEYGFLETPYRKV 600

*****************************************::**: *************

4YG2:C|PDBID|CHAIN|SEQUENCE TDGVVTDEIHYLSAIEEGNYVIAQANSNLDEEGHFVEDLVTCRSKGESSLFSRDQVDYMD 654

ST32 VDSKVTDQIDYLSAIEEGRYMIAQANAAIDEDGRLIDELVSSREAGETMMVTPDRIQYMD 660

.*. ***:*.********.*:*****: :**:*:::::**:.*. **: :.: *:::***

4YG2:C|PDBID|CHAIN|SEQUENCE VSTQQVVSVGASLIPFLEHDDANRALMGANMQRQAVPTLRADKPLVGTGMERAVAVDSGV 714

ST32 VAPSQIVSVAASLIPFLEHDDANRALMGSNMQRQAVPCLRPEKPVVGTGIERTCAVDSGT 720

*: .*:***.******************:******** ** :**:****:**: *****.

4YG2:C|PDBID|CHAIN|SEQUENCE TAVAKRGGVVQYVDASRIVIKVNEDEMYPGEAGIDIYNLTKYTRSNQNTCINQMPCVSLG 774

ST32 TVQAFRGGVVDYVDAGRIVIRVNDDEAVAGEVGVDIYNLIKYTRSNQNTNINQRPIVKMG 780

*. * *****:****.****:**:** **.*:***** ********* *** * *.:*

4YG2:C|PDBID|CHAIN|SEQUENCE EPVERGDVLADGPSTDLGELALGQNMRVAFMPWNGYNFEDSILVSERVVQEDRFTTIH**I**Q 834

ST32 DKVSRGDVLADGASTDLGELALGQNMLIAFMPWNGYNFEDSILISEKVVADDRYTSIH**I**E 840

: *.******** ************* :***************:**:** :**:*:***:

4YG2:C|PDBID|CHAIN|SEQUENCE ELACVSRDTKLGPEEITADIPNVGEAALSKLDESGIVYIGAEVTGGDILVGKVTPKGETQ 894

ST32 ELNVVARDTKLGPEEITRDISNLAEVQLGRLDESGIVYIGAEVEAGDVLVGKVTPKGETQ 900

** *:*********** ** *:.*. *.:************* .**:************

4YG2:C|PDBID|CHAIN|SEQUENCE LT**P**EEKLLRAIFGEKASDVKDSSLRVPNGVSGTVIDVQVFTRDGVEKDKRALEIEEMQLK 954

ST32 LT**P**EEKLLRAIFGEKASDVKDTSLRVPSGMSGTVIDVQVFTREGIQRDKRAQQIIDDELK 960

*********************:*****.*:************:*:::**** :* : :**

4YG2:C|PDBID|CHAIN|SEQUENCE QAKKDLSEELQILEAGLFSRIRAVLVAGG-------------VEAEKLDKLPRDRWLELG 1001

ST32 RYRLDLNDQLRIVEGDAFQRLARMLVGKVANGGPKKLAKGTKIDQAYLEDLDHYHWFDIR 1020

: : **.::*:*:*.. *.*: :**. :: *:.* : :*:::

4YG2:C|PDBID|CHAIN|SEQUENCE LTDEEKQNQLEQLAEQYDELKHEFEKKLEAKRRKITQGDDLAPGVLKIVKVYLAVKRRIQ 1061

ST32 LADDEAAASLEAIKNSIEEKRHQFDLAFEEKRKKLTQGDELPPGVLKMVKVYLAVKRRLQ 1080

*:*:* .** : :. :* :*:*: :* **:*:****:* *****:**********:*

4YG2:C|PDBID|CHAIN|SEQUENCE PGDKMAGRHGNK**G**VISKINPIEDMPYDENGTPVDIVLNPLGVPSRMNIGQILETHLGMAA 1121

ST32 PGDKMAGRHGNK**G**VVSKIVPIEDMPYMADGRPADVVLNPLGVPSRMNVGQVLEVHLGWAA 1140

**************:*** ******* :* *.*:************:**:**.*** **

4YG2:C|PDBID|CHAIN|SEQUENCE KGIGDKINAMLKQQQEVAKLREFIQRAYDLGADVRQKVDLSTFSDEEVMRLAENLRKGMP 1181

ST32 KGLGWRIGEMLQRQAKIEELRTFLTKIYNES---GRQEDLESFTDDEILELAKNLREGVP 1197

**:* :*. **::* :: :** *: : *: . :: **.:*:*:*::.**:***:*:*

4YG2:C|PDBID|CHAIN|SEQUENCE IATPVFDGAKEAEIKELLK----------LGDLPTSGQIRLYDGRTGEQFERPVTVGYMY 1231

ST32 FATPVFDGATEEEMGKMLDLAFPDDIAEQLGMNPSKNQVRLYDGRTGEMFERRVTLGYMH 1257

:********.* *: ::*. ** *:..*:********* *** **:***:

4YG2:C|PDBID|CHAIN|SEQUENCE MLKLNHLVDDKMHARSTGSYSLVTQQPLGGKAQFGGQRFGEMEVWALEAYGAAYTLQEML 1291

ST32 YLKLHHLVDDKMHARSTGPYSLVTQQPLGGKAQFGGQRFGEMEVWALEAYGASYVLQEML 1317

***:************* *********************************:*.*****

4YG2:C|PDBID|CHAIN|SEQUENCE T**V**KSDDVNGRTKMYKNIVDGNHQMEPGMPESFNVLLKEIRSLGINIELEDE 1342

ST32 T**V**KSDDVTGRTKVYENLVKGDHVIDAGMPESFNVLVKEIRSLGIDIDLDRN 1368

*******.****:*:*:*.*:* :: *********:********:*:*: :

**YedY**

1XDQ:A|PDBID|CHAIN|SEQUENCE --------------------------------------------------DLLSWFKGND 10

ST32 M**W**IKRPL**R**N**V**LTGAD**I**APSE**IT**P**R**AVFENRRRVLQAAGLAAAGGLFGTSGAALAAYA--- 57

*: :

1XDQ:A|PDBID|CHAIN|SEQUENCE RPPAPAGKALEFSKPAAWQNNLPLTPADKVSGYNNFYEFGLDKADPAANAGSLKTDPWT**L** 70

ST32 SPDARA-AKLAAKTNPTFVAIDKVTPFKDITSYNNFYEFGTDKSDPAQNAGTLRPRPWR**V** 116

* * * * .. :: :** ..::.******** **:*** ***:*: ** :

1XDQ:A|PDBID|CHAIN|SEQUENCE KISG**E**VAKPLTL**D**H**D**DLTRR**FP**LEER**I**YRMR**C**VE**A**WS**M**VVPW**I**G**FP**LH**K**LLALAE**P**TSNA 130

ST32 SVEG**E**VQHPKVF**D**L**D**ELLKL**AP**LEER**V**YRLR**C**VE**G**WS**M**VIPW**I**G**VP**LS**E**LIKRVQ**P**TGNA 176

.:.*:* :* .:* *:* : *****:**:****.****:****.** :*: .:**.**

1XDQ:A|PDBID|CHAIN|SEQUENCE KYVAFETIYAPEQMPGQQDRFIGGGLKYPYVEGLRLDEAM**H**PL**T**LMTVGVYG**K**ALPPQ**N**G 190

ST32 KYVQLVTLADPSQMPGLSTP----VLDWPYSEGLRMDEAM**N**PL**T**LLTMGVYG**Q**VLPNQ**N**G 232

*** : *: *.**** . *.:** ****:****:****:*:****:.** ***

1XDQ:A|PDBID|CHAIN|SEQUENCE **A**PVRLI**V**PWKYGFK**G**IKSIVSIKLTRERPPTTWNLAAPDEYGFYANVNPYVDHPRWSQ**A**T 250

ST32 **A**PVRIV**V**PWKYGFK**S**AKSLVKIRFVDKQPKTSWNTYAANEYGFYSNVNPNVDHPRWSQ**A**T 292

****::********. **:*.*::. ::* *:** * :*****:**** **********

1XDQ:A|PDBID|CHAIN|SEQUENCE ERFIGSGGILDVQRQPTLLFNGYADQV**A**S**L**YRG**LDL**RENFLEHHHHHH 298

ST32 ERRIGEDGFFTPK-RKTLMFNGYGDLV**A**S**M**YQG**MDL**KKNF-------- 331

** **..*:: : : **:****.* ***:*:*:**::**
