# Supplementary material for: What matters in chronic Burkholderia cenocepacia infection in cystic fibrosis: Insights from comparative genomics
Source: PLoS Pathog. 2017 Dec 11;13(12):e1006762. doi: 10.1371/journal.ppat.1006762 (PMC5739508; doi:10.1371/journal.ppat.1006762)
Supplement: S1 Fig — Each MLST sequence type is represented with one genome (see S2 Table). Complete genomes are denoted in bold. The ST32/ST33 lineage and the epidemic ET12 lineage are indicated. The tree was constructed from 95,357 variant nucleotide positions using the CSIPhylogeny pipeline. (PDF) [file ppat.1006762.s001.pdf]

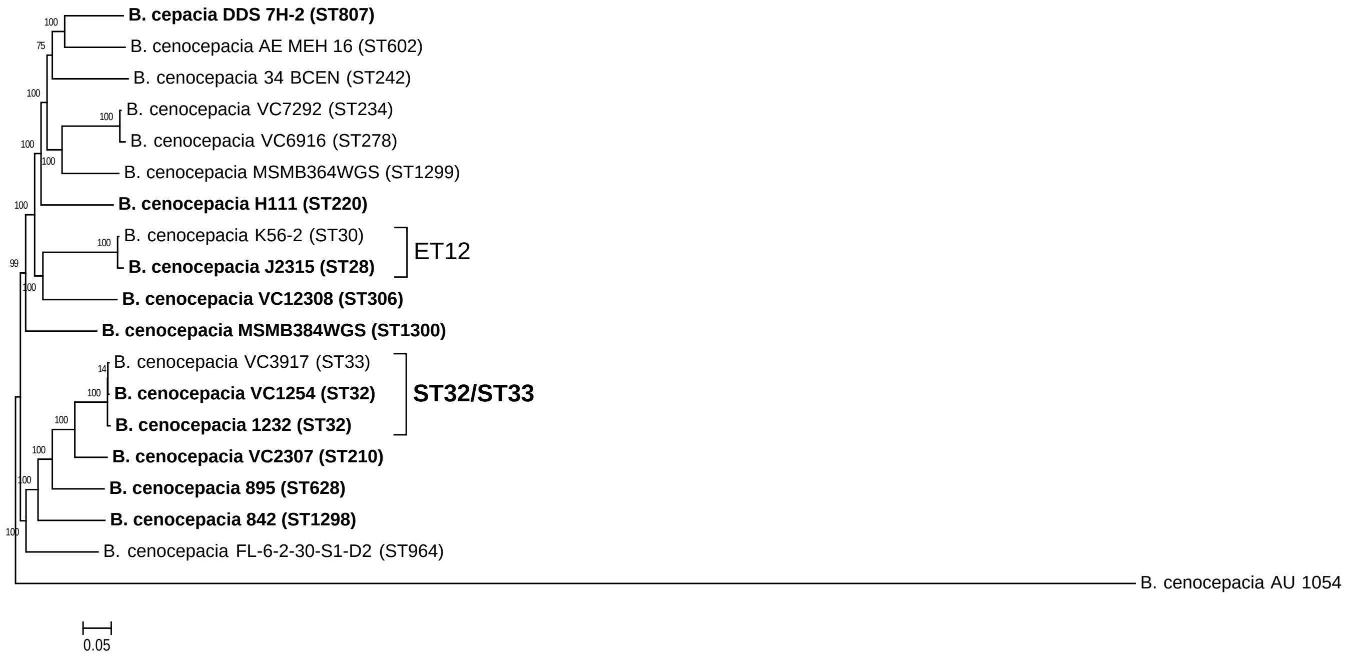
B. cepacia DDS 7H-2 (ST807)
B. cenocepacia AE MEH 16 (ST602)
B. cenocepacia 34 BCEN (ST242)
B. cenocepacia VC7292 (ST234)
B. cenocepacia VC6916 (ST278)
B. cenocepacia MSMB364WGS (ST1299)
B. cenocepacia H111 (ST220)
B. cenocepacia K56-2 (ST30)
B. cenocepacia J2315 (ST28)
ET12
B. cenocepacia VC12308 (ST306)
B. cenocepacia MSMB384WGS (ST1300)
B. cenocepacia VC3917 (ST33)
B. cenocepacia VC1254 (ST32)
B. cenocepacia 1232 (ST32)
ST32/ST33
B. cenocepacia VC2307 (ST210)
B. cenocepacia 895 (ST628)
B. cenocepacia 842 (ST1298)
B. cenocepacia FL-6-2-30-S1-D2 (ST964)
B. cenocepacia AU 1054
0.05
